# Multiple molecular defense strategies in *Brachypodium distachyon* surmount Hessian fly (*Mayetiola destructor*) larvae-induced susceptibility for plant survival

**DOI:** 10.1038/s41598-019-39615-2

**Published:** 2019-02-22

**Authors:** Subhashree Subramanyam, Jill A. Nemacheck, Andrea M. Hargarten, Nagesh Sardesai, Brandon J. Schemerhorn, Christie E. Williams

**Affiliations:** 1 Department of Agronomy, Purdue University, West Lafayette, IN 47907 USA; 2 USDA-ARS Crop Production and Pest Control Research Unit, West Lafayette, IN 47907 USA; 3 Corteva Agriscience, Agriculture Division of DowDuPont, Johnston, IA 50131 USA; 4 Department of Entomology, Purdue University, West Lafayette, IN 47907 USA

## Abstract

The Hessian fly is a destructive pest of wheat causing severe economic damage. Numerous genes and associated biological pathways have been implicated in defense against Hessian fly. However, due to limited genetic resources, compounded with genome complexity, functional analysis of the candidate genes are challenging in wheat. Physically, *Brachypodium distachyon* (Bd) exhibits nonhost resistance to Hessian fly, and with a small genome size, short life cycle, vast genetic resources and amenability to transformation, it offers an alternate functional genomic model for deciphering plant-Hessian fly interactions. RNA-sequencing was used to reveal thousands of Hessian fly-responsive genes in Bd one, three, and five days after egg hatch. Genes encoding defense proteins, stress-regulating transcription factors, signaling kinases, and secondary metabolites were strongly up-regulated within the first 24 hours of larval feeding indicating an early defense, similar to resistant wheat. Defense was mediated by a hypersensitive response that included necrotic lesions, up-regulated ROS-generating and -scavenging enzymes, and H_2_O_2_ production. Suppression of cell wall-associated proteins and increased cell permeability in Bd resembled susceptible wheat. Thus, Bd molecular responses shared similarities to both resistant and susceptible wheat, validating its suitability as a model genome for undertaking functional studies of candidate Hessian fly-responsive genes.

## Introduction

In recent years, the small grass species *Brachypodium distachyon* (Bd hereafter) has been extensively explored as a useful functional genomic model for grasses^1^. This genome offers advantages such as small size (diploid), short generation time, availability of vast genetic and genomic resources (T-DNA insertion mutant lines), and amenability to transformation^1,2^. These features make Bd an ideal model for functional genomics of candidate defense-response genes. In addition, Bd serves as a model system for nonhost resistance for several pathogens^3^, and insects^4,5^.

Hessian fly (*Mayetiola destructor*), a member of the gall midge family (order: Diptera, family: Cecidomyiidae), is an economically important destructive obligate pest of wheat (*Triticum aestivum*) causing significant yield loss^6^. After egg-hatch, the 1^st^ instar larvae crawl down to the base of the plant (crown) and attempt to establish sustained feeding sites. The wheat-Hessian fly interaction fits the gene-for-gene model^7^, yielding either incompatible or compatible interactions. During the incompatible interaction, Hessian fly resistance (*H*) gene-mediated plant defense results in larval death within 4–5 days after egg-hatch (DAH) allowing the plant to be resistant and grow normally^8^ after some initial growth penalties^5^. In contrast, in the compatible interaction, within 3 days of larval attack, the larvae alter host metabolic pathways^9^ resulting in the formation of a nutritive tissue^10^ providing the larvae a nutrient-rich diet. The virulent larvae utilize these plant-derived nutrients^11^ allowing them to develop and complete their life cycle while the growth of the susceptible wheat host is stunted^12^.

Deployment of Hessian fly resistance (*H*) genes is the most effective way to manage this insect pest. To date 35 *H (H1* to *H34* plus *Hdic*) genes have been identified^13–15^. However, the use of *H* gene resistance results in the selection of Hessian fly biotypes that can overcome deployed resistance posing a great challenge to the long-term protection of wheat. The identification and functional characterization of defense-related, downstream genes responsive to Hessian fly feeding allow us to exploit an alternate pathway in the management of this pest. These genes could be utilized for complementing native or introgressed *H* gene resistance in wheat, thereby prolonging durability of a wheat cultivar in the field.

Gene expression studies, using Affymetrix wheat arrays and quantitative real-time PCR techniques, have been undertaken and several Hessian fly-responsive genes associated with resistance and susceptibility have been identified^16–23^. Expression analyses revealed differential regulation of genes encoding defense response proteins including lectins, dirigent proteins, regulatory proteins (protein kinases and transcription factors), and secondary metabolites thus highlighting putative roles played by these molecules in wheat defense against Hessian fly. Genes encoding enzymes involved in polyamine biosynthesis pathway, heat shock proteins, and pore-forming toxins were differentially expressed during susceptibility. However, further functional analyses of these defense proteins through supplementation and/or mutational approaches are challenging due to the complexity of the wheat genome, and limited genetic and genomic resources. This necessitates the use of model systems, such as Bd, which displays nonhost Hessian fly resistance, to undertake downstream functional studies. Additionally, Bd and wheat diverged from a common ancestor very recently, and hence share highly conserved sequence homology and gene synteny^24^.

As a first step towards developing Bd as a functional surrogate genome model for wheat-Hessian fly interactions, we recently characterized the physical and metabolic responses of Bd (Line 21) plants to Hessian fly attack^5^. Bd plants, when challenged with Hessian fly, exhibited type I and type II nonhost resistance and, similar to the resistant host wheat, showed no yield penalty. However, unlike the resistant host wheat, around 25% of the larvae continued to grow through 20 days after egg-hatch with some surviving up to 41 days longer than the avirulent larvae feeding on resistant wheat, and 3 weeks longer than required for virulent larvae to pupate on susceptible wheat^5^. However, despite the extended period of survival, the larvae growing on Bd plants failed to complete their development. In addition, Hessian fly-infested Bd plants showed presence of necrotic lesions, indicating a robust hypersensitive response. Transcript profiling of a few Hessian fly-responsive marker genes revealed expression profiles that shared similarities with both the resistant and susceptible wheat^5^. Thus, Bd plants exhibited physical and metabolic characteristics intermediate to both resistant and susceptible host wheat in response to Hessian fly larval attack.

To successfully utilize Bd as a model genome for functional analyses of candidate genes responsive during wheat-Hessian fly interactions, it is essential to dissect the entire transcriptome of nonhost Bd to reveal genes and associated biological pathways responsive to Hessian fly that are common or unique to host wheat. Deep-sequencing technologies, such as RNA-sequencing (RNA-seq), offer a powerful tool not only to discover new genes and transcripts but also to measure transcript expression in a single assay. In the current study, transcriptome analysis of Bd infested with Hessian fly larvae, undertaken using high throughput RNA-seq, revealed thousands of differentially expressed genes (DEGs) over a time-course. Results clearly revealed that Bd molecular responses shared similarities to both resistant and susceptible wheat, validating its suitability as a model genome for undertaking downstream functional studies of candidate Hessian fly-responsive genes associated with wheat resistance and susceptibility.

## Results and Discussion

Our previous study revealed that the model grass genome, *Brachypodium distachyon* (Bd21)^1^, exhibits nonhost resistance to Hessian fly attack and shares similarities at the physical and metabolic levels to wheat host-plant resistance and susceptibility^5^. These findings offer avenues for utilizing the recently sequenced well-annotated Bd21 model genome^24^, and the large collections of available mutant knockout lines generated in this background^2^ for functional characterization of candidate genes that putatively play a role in host wheat defense and susceptibility to Hessian fly. To gather information about the transcriptional events occurring in Bd plants in response to Hessian fly larval feeding, RNA-seq analysis was performed using crown tissue (feeding site) from Hessian fly-infested Bd plants (Supplementary Fig. S1**)** over a time-course of 1 DAH (Bd1), 3 DAH (Bd3), and 5 DAH (Bd5) and compared to that of uninfested control Bd plants at 1 DAH (BdC).

### RNA-seq data analyses

**Figure 1:**
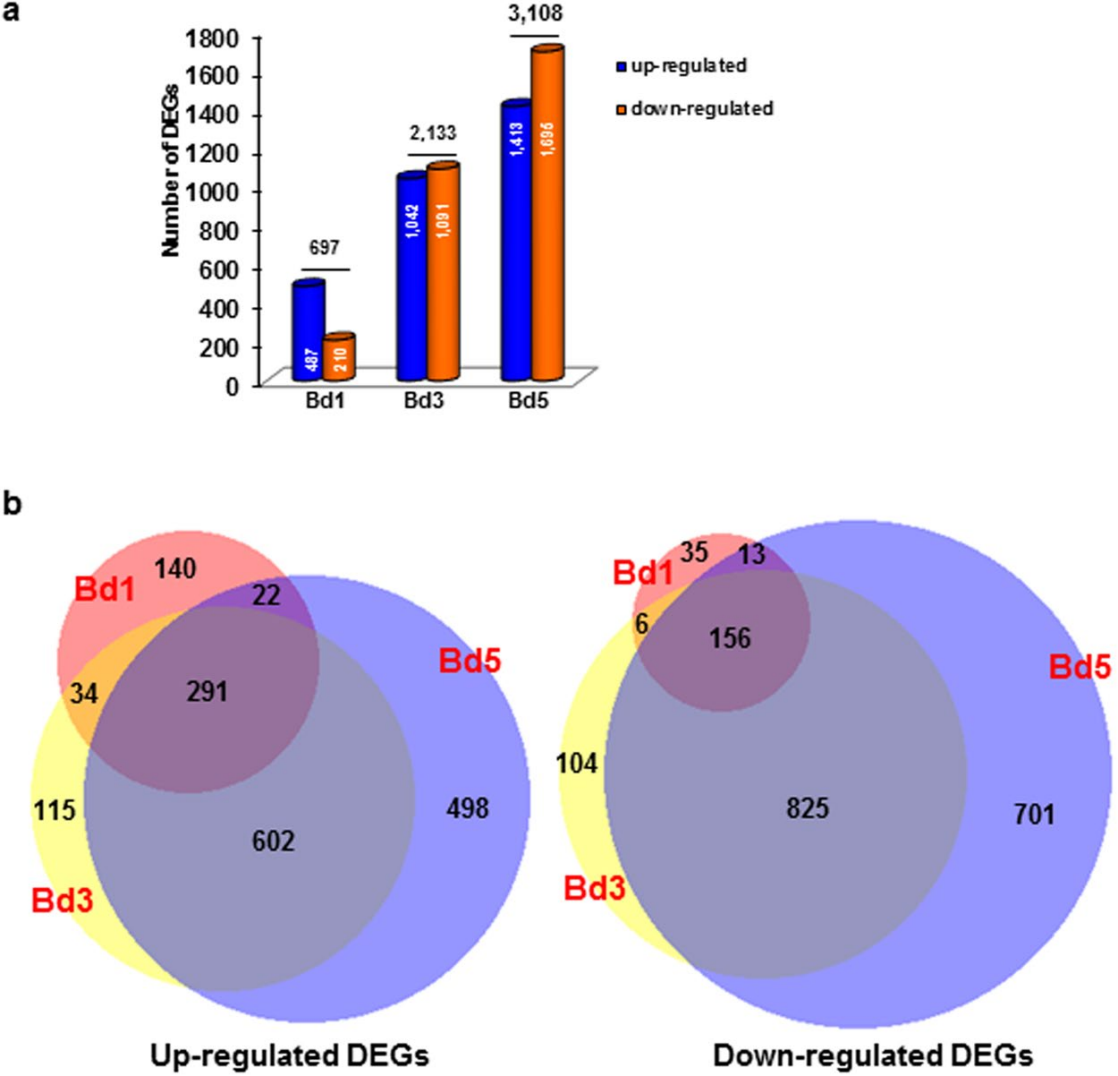
Differential expression of *Brachypodium* genes in response to Hessian fly infestation. Differentially expressed genes (DEGs) in Hessian fly-infested Bd plants were identified over a time-course of 1 (Bd1), 3 (Bd3) and 5 (Bd5) days after egg-hatch (DAH) as compared to the uninfested control plants. (**a**) Number of statistically significant (>2-fold change, *p* < 0.05) up-regulated and down-regulated DEGs in Bd samples. Number of genes for each category are indicated (**b**) Venn diagram depicting shared and uniquely expressed up-regulated (left panel) and down-regulated (right panel) DEGs in Bd1 (orange circle), Bd3 (yellow circle), and Bd5 (blue circle).

A total of 473,109,838 reads were acquired, ranging from 27 to 63 million reads for each transcriptome (Table 1). More than 70% of the filtered reads successfully mapped to the Bd21 reference genome sequence with a significant fraction of reads being unique and limited number of multi-mapping reads (Table 1). Hessian fly infestation led to significant differential expression (≥2-fold-change, *p* < 0.05) of 3,067 transcripts in Bd1 (487 up-regulated; 210 down-regulated), Bd3 (1,042 up-regulated; 1091 down-regulated), and Bd5 (1,413 up-regulated; 1,695 down-regulated) samples (Fig. 1a), with the samples at three time-points sharing some unique and common DEGs (Fig. 1b). Although fewer number of DEGs were observed in Bd1 samples, there were more up-regulated than down-regulated genes. In Bd3, similar numbers of up- and down-regulated DEGs were observed. However, in Bd5, the number of down-regulated genes were relatively higher than up-regulated genes (Fig. 1a). Of the up-regulated genes, 291 genes were common between Bd1, Bd3 and Bd5, while 156 down-regulated genes were common between the three time-points (Fig. 1b). Gene ontology analysis assigned 3,065 out of 3,067 DEGs to 26 GO terms for the domains of biological process, cellular component, and molecular function (Supplementary Fig. S2). The majority of transcripts assigned to the ‘biological process’ domain were involved in metabolic (GO:0008152) and cellular activities (GO:0009987). Highest number of transcripts under ‘molecular function’ domain belonged to catalytic (GO:0003824), followed by binding (GO: 005488), and transporter (GO:0006810) functions. A significant enrichment of genes in GO domains involved in catalytic, metabolic, and cellular process in infested Bd plants suggests molecular perturbations of the nonhost in response to Hessian fly larval attack.

**Table 1:** Summary of RNA-seq reads and mapping data using the Bd21 annotated reference genome.

Sample name*	Total reads	Mapped reads	Total Mapped (%)	Uniquely mapped (%)	Multi-mapped (%)
BdC-1	317,593,78	266,765,22	84.00	59.66	24.34
BdC-2	376,231,98	319,671,32	84.97	60.05	24.91
BdC-3	639,463,99	473,519,01	74.05	52.90	21.15
Bd1-1	414,037,15	318,540,32	76.94	54.33	22.61
Bd1-2	440,317,09	343,637,36	78.04	54.57	23.47
Bd1-3	419,353,66	330,276,71	78.86	54.47	24.29
Bd3-1	276,809,69	217,011,70	78.40	54.53	23.87
Bd3-2	329,727,73	258,806,63	78.49	54.82	23.67
Bd3-3	326,091,84	266,586,62	81.75	55.60	26.13
Bd5-1	302,618,50	243,412,27	80.44	54.68	25.76
Bd5-2	550,466,85	441,756,54	80.25	54.63	25.63
Bd5-3	338,386,22	253,805,98	75.00	52.27	22.73

*The numbers 1, 2 and 3 at the end of the sample name represent the three biological replicates.

### qRT-PCR validates RNA-seq expression results

Fifteen representative DEGs (9 up-regulated and 6 down-regulated) were selected to carry out qRT-PCR expression studies to validate the RNA-seq expression data. The RNA-seq expression data were highly correlated with the qRT-PCR expression results (Supplementary Table S1), and the Pearson correlation coefficient for the expression results between the two methods was 0.94 (Supplementary Fig. S3) confirming the validity of the RNA-seq data.

### Diverse biotic stress pathways are involved in Bd response to Hessian fly attack

MapMan pathway analysis tool highlighted key biotic stress pathways responsive in Bd plants following Hessian fly larval attack. This analysis revealed significant up-regulation of several pathways documented to play a role in plant defense including redox metabolism, signal transduction, secondary metabolites, signaling hormones, and heat shock proteins as early as 1 DAH (Bd1 sample), thus indicating an early defense strategy employed by Bd plants (Supplementary Fig. S4a). Although, by later time-points transcripts for several of these defense pathway genes were still increased, a significant number of genes were also down-regulated (Supplementary Fig. S4b,c) indicating a counter defense by the larvae to suppress these defense pathways. Most cell wall-associated genes were significantly down-regulated in all three samples (Supplementary Fig. S4) suggesting that the larvae may try to overcome cell wall barriers. Each biotic stress pathway was further dissected to reveal the specific subset of genes, gene families, and their expression profiles in response to Hessian fly larval feeding.

### Differential expression of defense-related proteins

**Figure 2:**
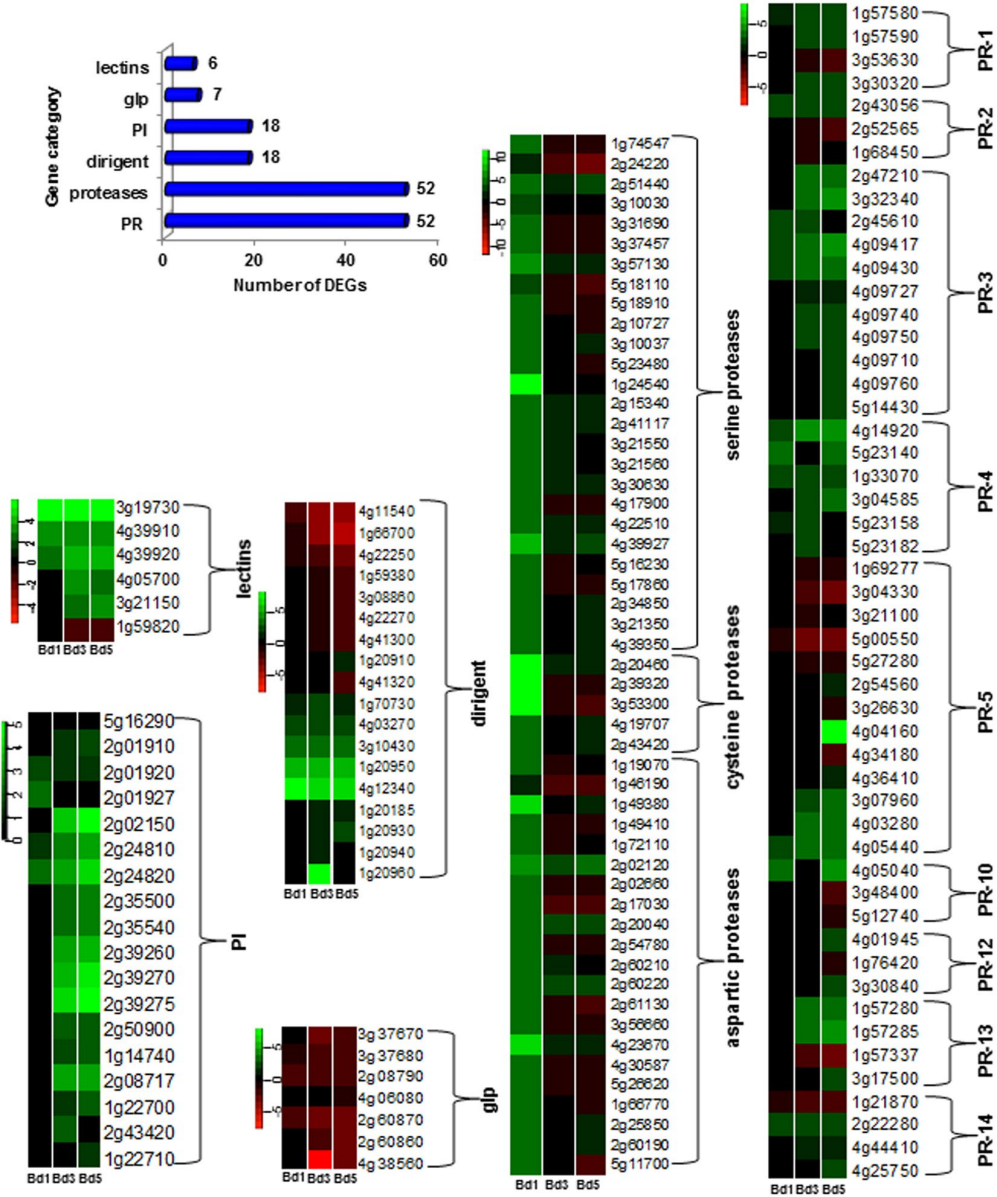
Differential expression of defense-related genes in *Brachypodium* infested with Hessian fly. Number of DEGs encoding several classes of defense-related proteins represented in the Bd transcriptome are indicated in the chart. Heatmaps depict expression profiles of genes encoding defense-related proteins over a time-course of 1 (Bd1), 3 (Bd3), and 5 (Bd5) DAH. All DEGs belonging to one class of defense-related proteins are grouped together within a heatmap. Since all genes represent the Bd Gene-IDs, the prefix “Bradi” has been removed and only the number associated with a particular Gene-ID is given for identification. Green represents up-regulated genes and red represents the down-regulated genes, while genes not differentially expressed at a particular time-point are indicated in black. glp: germin-like proteins, PI: protease inhibitors, PR: pathogenesis-related.

In response to insect attack, plants protect themselves by activating defense responses via various morphological, biochemical, and molecular mechanisms, with a crucial component being the expression of defense-related (DR) proteins^25,26^. Several classes of DR proteins, including lectins, pathogenesis-related (PR) proteins, protease inhibitors (PI), and proteases have been identified that counter a pathogen/pest^27,28^. RNA-seq of Bd plants revealed DEGs encoding lectins (6), germin-like proteins (glp, 7), proteases (52), PI (18), dirigent (18), and PR proteins, (52) following Hessian fly larval feeding (Fig. 2). Evidence of increased defense in Bd comes from elevated expression of lectins (Supplementary Table S2, Fig. 2) which are glycoproteins having insecticidal properties against several insects^29^. Two Hessian fly-responsive genes, *Hfr-1* and *Hfr-3* (*Hessian fly-responsive gene 1* and *3*), encoding mannose-binding jacalin-like and chitin-binding lectins, respectively, were strongly up-regulated in resistant wheat^17,18^. In fact, HFR-1 and HFR-3 proteins have insecticidal and antifeedant properties against *Drosophila melanogaster*^30^, and cereal aphid pest, *Sitobion avenae*^31^. The strong up-regulation of lectins in Bd suggests a likely defense role in Hessian fly antibiosis. Similar to lectins, dirigent-like proteins that are strongly up-regulated during wheat resistance to Hessian fly^21^ were also differentially expressed in Bd (Fig. 2). Transcripts for all types of PIs were also significantly increased by Bd3 and Bd5 time-points (Supplementary Table S2, Fig. 2), which may potentially function by targeting gut proteases, and prevent the larvae from developing normally, as observed in other plant-insect interactions^32,33^. Alternatively, plant cell wall-damaging proteases that are present in salivary secretions of the Hessian fly larvae^34^ could be neutralized by the Bd PIs as a primary defense mechanism. Salivary proteases that may have a role in pre-oral digestion of food have been reported in other insect species such as the wheat midge (*Sitodiplosis mosellana*)^35^ and the rice brown planthopper (*Nilaparvata lugens*)^36^. Although, germins and glps are involved in hydrogen peroxide (H_2_O_2_) and ethylene production and function in defense against insect herbivores^37,38^, in Hessian fly-infested Bd plants glps were significantly down-regulated at all time-points (Fig. 2) and may not play a defense role. Several proteases were up-regulated in the Bd3 and Bd5 samples (Fig. 2), indicating a defense strategy against Hessian fly. Plant insecticidal proteases target the midgut, peritrophic matrix, hemocoel, and cuticle of the herbivorous insects^39^. However, decreased levels of certain plant proteases, such as aspartic proteases, by the later stages in Bd3 and Bd5 samples (Fig. 2) suggest that the larvae are able to attenuate the defense responses in an attempt to survive as they establish feeding sites. In fact, 29 distinct PIs have been identified in Hessian fly larval gut and salivary glands that are expressed at different stages in the growth of the larvae and may play a role in protecting the insect from plant-derived proteases^40^. PR proteins either affect herbivore development^41^ or trigger plant defensive barriers^42^. Based on their primary structure and biological activity they are classified into different families designated as PR-1 to PR-17^43^. Bd RNA-seq analysis revealed several DEGs encoding PR proteins belonging to different families including PR-1 to 5, PR-10, and PR-12 to 14 (Fig. 2). Expression for most members of these PR families only changed in the Bd3 and Bd5 time-points (Fig. 2). Most genes encoding PR-3 (chitinase), PR-4 (wound induced protein), PR-13 (thionin), and PR-14 (lipid transfer protein) were up-regulated. Six of thirteen genes encoding PR-5 (osmotin and thaumatin-like proteins) were up-regulated, but mostly in later time-points of Bd3 and Bd5 samples, while the rest were down-regulated (Supplementary Table S2). Our results indicate the involvement of a classical PR response in nonhost Bd defense to Hessian fly, similar to plant-pathogen interactions, but in contrast to host wheat, that lacks a traditional PR defense response^13,44^. PR proteins are documented to be more common in resistance responses linked to a hypersensitive response (HR). Since Bd plants, unlike most host wheat, are shown to develop HR-like symptoms^5^ it is plausible that the PR protein-linked HR initiates a resistance mechanism.

### Regulation of genes encoding signaling hormones

Phytohormones such as salicylic acid (SA), jasmonic acid (JA), ethylene (ET), abscisic acid (ABA), auxins (AUX), cytokinins (CK), and gibberellic acid (GA) play crucial roles in defense signaling in addition to being critical factors in regulating plant growth and development^45,46^. These signaling pathways cross-talk in either an antagonistic or synergistic manner forming a complex network that provides plants the regulatory potential to adapt to rapidly changing biotic environments^45^. The Bd transcriptome dataset revealed DEGs involved in the biosynthesis of select phytohormones (Supplementary Fig. S5). Except for a few DEGs at certain time-points, most genes in AUX and GA pathways were down-regulated, with around half in the ABA pathway being up-regulated late, and the other half being down-regulated (Supplementary Table S3). As auxins and GA play an important role in cell elongation impacting plant growth and development^45^, down-regulation of these hormones may have a direct role in causing the stunted phenotype of Bd plants infested with Hessian fly (Supplementary Fig. S1). ET pathway was represented by only two genes with one being up-regulated early and other down-regulated late. The 3-fold up-regulated gene (*Bradi2g41840*) was annotated as aminocyclopropane carboxylate (ACC) oxidase. ACC oxidase is up-regulated in aphid-susceptible celery and *Arabidopsis* infested with *Myzus persicae*^47^. Genes associated with SA signaling pathway, were not differentially expressed in Hessian fly-infested Bd plants. Biosynthesis of SA in plants takes place through two potential pathways: isochorismate and the phenylalanine ammonia-lyase (PAL) pathway from trans-cinnamic acid^48,49^. In *Arabidopsis*, SA synthesis occurs primarily through the isochorismate pathway via the activity of isochorismate synthase^49^. There was no change in the transcript levels of *Bradi4g28670* encoding isochorismate synthase, in Bd plants infested with Hessian fly (Supplementary Table S3). In the PAL pathway, of the seven PAL homologs identified in Bd (*Bradi5g15830*; *Bradi3g48840*; *Bradi3g49280*; *Bradi3g49260*; *Bradi3g47110*; *Bradi3g47120*; *Bradi3g49250*) only *Bradi3g48840* was up-regulated 4-fold in Bd1 samples. However, none of the other genes downstream of PAL involved in biosynthesis of SA such as those encoding 4-coumarate:CoA ligase (homologs *Bradi3g37300*; *Bradi3g05750*; *Bradi1g31320*) and aldehyde oxidase (homologs *Bradi1g52740*; *Bradi1g56667*; *Bradi1g06177*) showed differential expression, suggesting that the products of the PAL activity are funneled towards the biosynthesis of secondary metabolites instead of towards SA-mediated defense responses. Several genes involved in JA pathway were up-regulated, except for lipoxygenase which was down-regulated, suggesting that Bd resistance to Hessian fly follows the JA-mediated defense, more closely associated with resistance to herbivorous insects^50^. In fact, JA and ET co-regulate the expression of *PR-3*, *PR-4* and *PR-12* genes^51^ that were up-regulated in Bd. All DEGs involved in the CK signaling pathway were strongly up-regulated in Bd, with the transcript abundance increasing from 1 to 5 DAH time-points. Increased CK concentrations following pathogen or insect attack are postulated to play a role by reconfiguring primary and secondary metabolism associated with plant-induced defense^52^. In several insect species CK mediate plant resistance through production of secondary metabolites that deter insect feeding, delay larval development, or reduce weight gain by the insect^53^. However, galling insects, similar to Hessian fly, are associated with the induction of green islands, and/or the modification of source-sink relationship leading to nutrient translocation towards the insect’s feeding site. Increased CK levels in the infested tissue are proposed to stimulate growth of nutrient-rich green islands induced by galling insects^52^. Unlike other galling insects Hessian fly does not form a typical gall, but reprograms the susceptible wheat resulting in differentiation of a gall-like nutritive tissue that is rich in nutrients^10^. Thus, increased CK in Bd plants during the later time-points may be a strategy employed by the larvae to induce a nutrient-rich tissue as a possible source of nutrition, not associated with plant defense, as observed in other plant-insect interactions.

### Genes involved in reactive oxygen species production and scavenging

Plants counter biotic stress by rapid production of reactive oxygen species (ROS) radicals, referred to as ‘oxidative burst’. This leads to a HR resulting in a zone of cell death around the area of the stress^54^. ROS accumulation can: (i) directly kill the pathogen cells, (ii) cross-link plant cell wall proteins for reinforcement, (iii) signal up-regulation of DR proteins, or (iv) trigger plant cell death that restricts pathogen growth^55^. Another indication of occurrence of an oxidative burst in plants is the increased transcript level of genes encoding enzymes involved in ROS-scavenging process, that limits ROS damage to the host cells^56^.

Despite HR, as a plant defense, being strongly associated with gene-for-gene model in plant-pathogen interactions, it is not a pre-requisite during wheat-Hessian fly interactions. In fact, most resistant wheat lines lack a visible HR. However, HR-like lesions have been observed in the wheat lines ‘Flynn’^57^ and ‘Hamlet’ (S.S. & J.A.N., unpublished data) harboring the *H6* and *H21* resistance genes, respectively. Giovanini *et al*.^58^ noted the lack of a classical oxidative burst mediated by NADPH-dependent oxidase (NOX) during wheat-Hessian fly interactions in the wheat line ‘*H9*-Iris’. However, Liu *et al*.^55^ proposed the involvement of class III peroxidases (POX) instead of the classical NOX-mediated oxidative burst mechanism in wheat as well as nonhost rice plants in response to Hessian fly attack.

**Figure 3:**
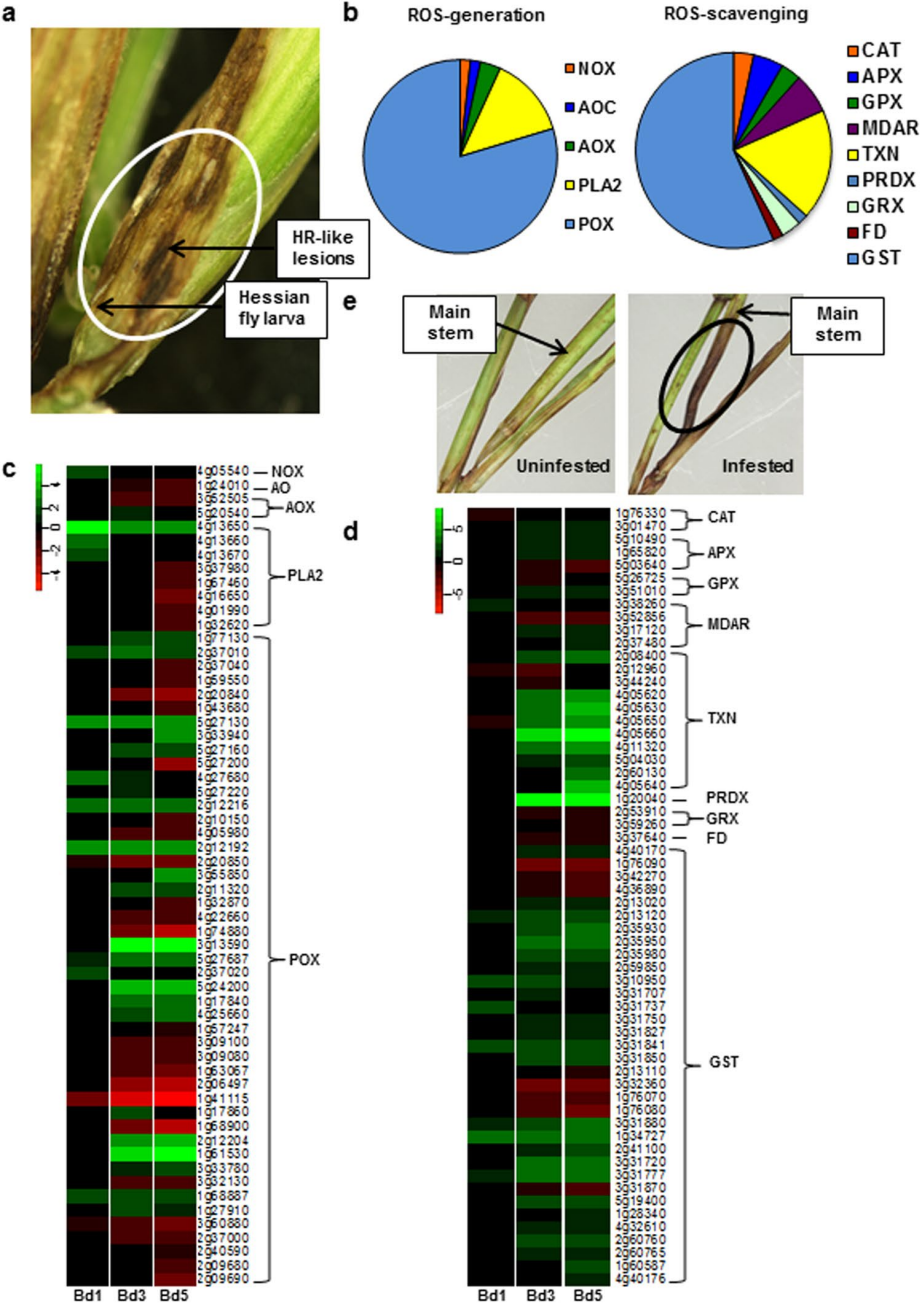
Differential regulation of reactive oxygen species (ROS) metabolism genes in *Brachypodium* infested with Hessian fly. (**a**) Bd plants exhibiting dark hypersensitive response (HR)-like necrotic lesions on the main stem. (**b**) Pie charts showing DEGs encoding ROS-generating (left chart) and -scavenging (right chart) enzymes represented in the Bd transcriptome. (**c**,**d**) Heatmaps depicting the expression profiles of enzymes involved in ROS-generation (**c**) and -scavenging (**d**) over a time-course of 1 (Bd1), 3 (Bd3), and 5 (Bd5) DAH. Since all genes represent the Bd Gene-IDs, the prefix “Bradi” has been removed and only the number associated with a particular Gene-ID is given for identification. Green represents up-regulated genes and red represents the down-regulated genes, while genes not differentially expressed at a particular time-point are indicated in black. (**e**) Hessian fly-infested Bd plant (right panel) showing positive (brown coloration) DAB (3,3′-diaminobenzidine) staining at 1 DAH confirming ROS accumulation. Uninfested Bd plants (left panel) were used as controls for the DAB staining. NOX: NADPH-dependent oxidase, AO: amine oxidase, AOX: alternative oxidase, PLA2: phospholipase A2, POX: class III peroxidase, CAT: catalase, APX: ascorbate peroxidase, GPX: glutathione peroxidase, MDAR: dehydroascorbate reductase, TXN: thioredoxin, PRDX: peroxiredoxin, GRX: glutaredoxin, FD: ferrodoxin, GST: glutathione-S-transferase.

Hessian fly-infested Bd plants exhibited visible HR-like symptoms with large, darkened necrotic lesions present on the main stems (Fig. 3a), as documented previously^5^. At the transcriptome level, Bd plants also showed differential regulation of genes encoding ROS-generating and -scavenging enzymes (Fig. 3b). The ROS-generating enzymes included NOX, amine oxidase (AO), alternative oxidase (AOX), phospholipase (PLA2), and POX (Fig. 3b). A NOX-encoding gene, *Bradi4g05540*, was significantly up-regulated in Bd 1 sample (2.8-fold; *p* < 0.001) but showed no change in Bd3 and Bd5 samples (Supplementary Table S4). These results implicate NOX-mediated ROS accumulation in Bd plants, unlike host wheat^55,58^. Genes encoding AOX and AO proteins were down-regulated. Although there were many genes encoding PLA2 and POX that were significantly down-regulated, there were a few that were up-regulated with very high expression (Fig. 3c). For example, *Bradi4g13650* encoding PLA2 was up-regulated 7–43 fold (*p* < 0.00001), and *Bradi5g27130* encoding POX was up-regulated 8–9 fold (*p* < 0.00001) in all three time-points, similar to observations in Hessian fly-infested host wheat^55^. ROS accumulation in host cells is extremely harmful, hence plants produce several scavenging enzymes^56^. Our RNA-seq data showed accumulation of transcripts encoding catalase (CAT), peroxiredoxin (PRDX), thioredoxin (TXN), and glutathione-S transferase (GST), classically involved in the ROS scavenging process (Fig. 3d), indicating the generation of ROS and their subsequent detoxification in Hessian fly-infested Bd plants. The mRNA abundance for a gene encoding GST (*Bradi3g31777*) was as high as 11-fold, and that encoding PRDX (*Bradi1g20040*) was 232-fold. Additionally, histochemical staining with 3,3′-diaminobenzidine (DAB) stain showed intense brown staining of the crown of the main stem (harboring maximum number of larvae) in the infested Bd plants, indicating presence of H_2_O_2_, a ROS radical (Fig. 3e). The HR symptoms, increased transcripts for ROS-generating and -scavenging enzymes, and positive DAB staining, put together, clearly support the involvement of ROS in Bd defense against Hessian fly.

While a great deal of evidence exists for the role ROS and HR play in plant defense against pathogens^59^, their role in insect defense remains unclear. HR has been documented to be an effective resistance strategy against insects that are immobilized within their gall, like those observed in the gall midge *Schizomyia macrocapillata* feeding on *Bauhinia brevipes*, where necrosis around the gall kills 90% of the larvae^60^. However, Hessian fly larvae feeding on Bd are mobile and migrate away from the necrotic lesions to healthy green tissue (base of the leaf)^5^ suggesting that larval development may not be directly affected by HR. However, in addition to direct effects, ROS can also induce an array of cellular protection mechanisms, thus functioning as secondary messengers for activating defense response genes^61^. H_2_O_2_ activates basic PR proteins^62^ and ROS inhibition by diphenylene iodonium, results in blocked activation of DR genes and accumulation of phytoalexins^61^. The increased ROS-generation observed in Bd in response to Hessian fly larval attack could plausibly be playing an indirect defense role, instead of a direct role as observed in plant-pathogen interactions. The increased transcripts of PR proteins and select signaling hormones (Supplementary Tables S2 and S3) in Bd plants may be correlated with increased ROS activity. Similar increase in expression of PR proteins, as well as genes involved in hormone signaling pathways was observed in Hessian fly-infested host wheat showing HR (S.S. & J.A.N., unpublished data), although it was not observed in infested wheat lacking HR^13^.

### Expression of heat shock proteins in Bd plants

Heat shock proteins (HSPs) are ubiquitous and induced in response to a wide array of abiotic and biotic stresses. These proteins contribute to cellular homeostasis, perform critically important chaperone functions, and assist in the refolding of proteins within cells damaged by stress^63^. Based on their molecular weights, HSPs are classified into five major families in plants^63^. Hessian fly-infested Bd revealed DEGs representing all the 5 families (Supplementary Fig. S6), HSP100 (2), HSP90 (3), HSP70 (4), HSP60 (1), and small HSP (sHSP, 23). HSP90, HSP70 and HSP60 were highly up-regulated. Short-term oxidative stress treatment resulted in increased transcripts of HSP-encoding genes in *Arabidopsis*^64^, suggesting that the increased HSP transcripts observed in Bd transcriptome may be regulated by the increased oxidative stress discussed above (Fig. 3). Several of the sHSPs were also up-regulated as high as 68-fold in Bd5 (Supplementary Table S5). We speculate that while HSP90, HSP70 and HSP60 may be playing a role in plant defense, the strong induction of some sHSPs in response to Hessian fly infestation may be involved in the suppression of resistance in Bd, resembling the expression profile of *Mds-1* (*Mayetiola destructor susceptibility-1*), a gene induced in susceptible host wheat that suppresses *H*-gene mediated resistance^22^. *Mds-1* encodes a sHSP which when silenced confers immunity to normally susceptible host wheat against all Hessian fly biotypes. The Bd transcriptome revealed homologs of wheat *Mds-1* in the dataset, *Bradi2g02400* and *Bradi2g02410*, that were strongly up-regulated 68- and 43-fold, respectively. Similar to susceptible wheat, the sHSP induced in Bd may be involved in the attempted suppression of nonhost resistance to Hessian fly.

### Secondary metabolites as key defense molecules in Bd plants

**Figure 4:**
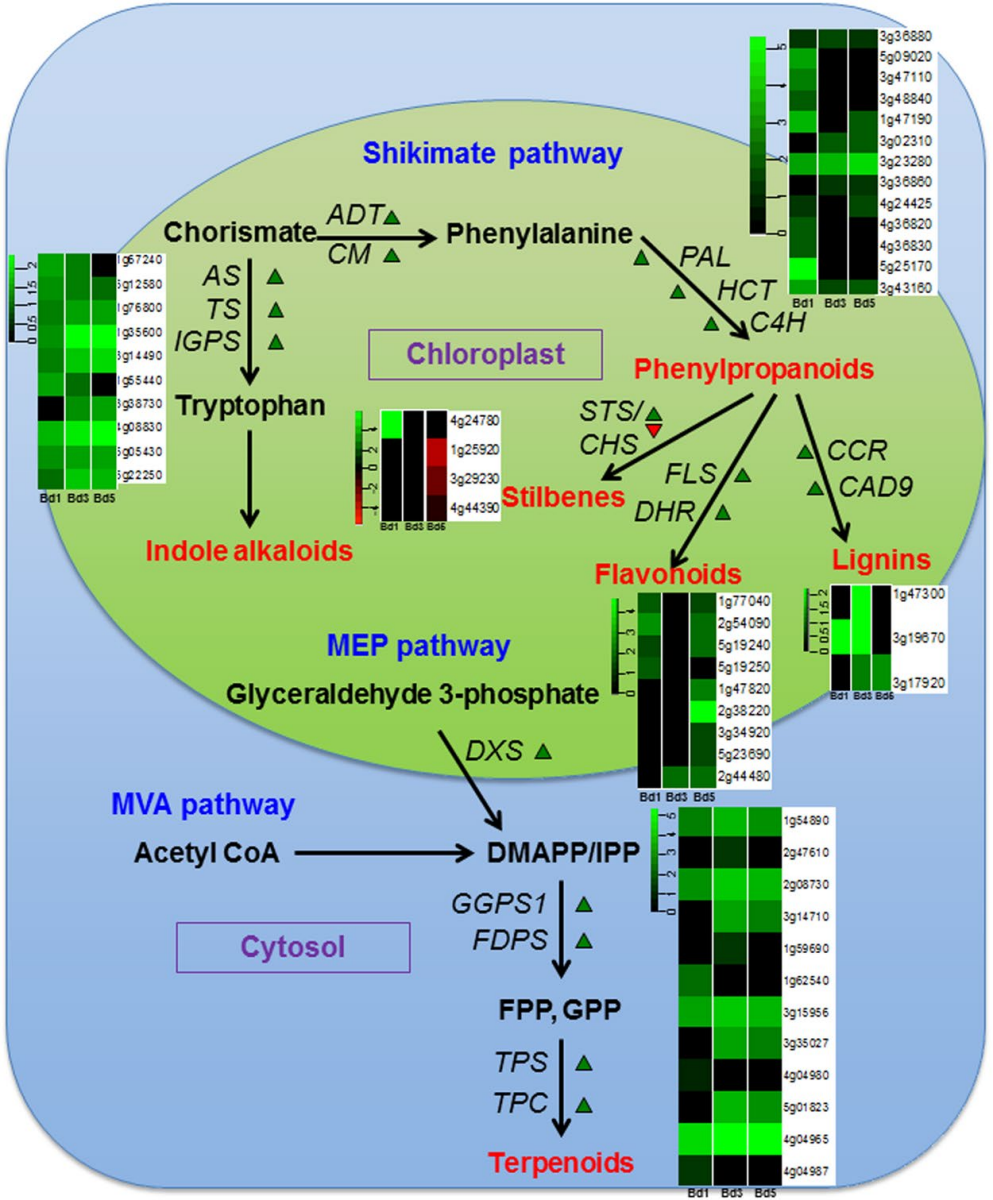
Differential regulation of *Brachypodium* genes involved in the biosynthesis of secondary metabolites in response to Hessian fly larval attack. Schematic representation of secondary metabolites produced in plants via Shikimate, MVA (mevalonic acid), and MEP (methylerythritol phosphate) pathways. The pathways are localized to the chloroplast and the cytosol. The DEGs involved in the production of secondary metabolites are displayed. Green arrows denote up-regulated and red arrows denote down-regulated genes. Heatmaps depict the expression profiles of the DEGs over a time-course of 1 (Bd1), 3 (Bd3), and 5 (Bd5) DAH, with all genes producing one type of secondary metabolite clustered together. Since all genes represent the Bd Gene-IDs, the prefix “Bradi” has been removed and only the number associated with a particular Gene-ID is given for identification. On the heatmaps, green represents up-regulated genes and red represents down-regulated genes, while genes not differentially expressed at a particular time-point are inducated in black. AS: anthranilate synthase, TS: tryptophan synthase, IGPS: indole-3-glycerol phosphate synthase, ADT: arogenate dehydratase, CM: chorismate mutase, PAL: phenylalanine ammonia lyase, HCT: hydroxycinnamoyl-CoA shikimate/ quinate hydroxycinnamoyl transferase, C4H: cinnamate-4-hydroxylase, STS: stilbene synthase, CHS: chalcone synthase, FLS: flavonol synthase, DHR: dihydroflavonol 4-reductase, CCR: cinnamoyl CoA reductase, CAD: cinnamyl alcohol dehydrogenase, DXS: deoxyxylulose-5-phosphate synthase, GGPS: geranylgeranyl pyrophosphate synthase, FDPS: farnesyl diphosphate synthase, TPS: terpene synthase, TPC: terpenoid cyclase, DMAPP/IPP: dimethylallyl pyrophosphate/isopentenyl diphosphate, FPP: farnesyl pyrophosphate, GPP: geranylgeranyl pyrophosphate.

Plant secondary metabolites play an important role with respect to their induced defense properties in response to insect herbivores^65^. They are categorized into three main groups based on their biosynthetic origin: (i) alkaloids, (ii) phenylpropanoids, and (iii) terpenoids. While alkaloids and phenylpropanoids are produced through the shikimate pathway, the terpenoids are derived from the mevalonic acid (MVA) and methylerythritol phosphate (MEP) pathways, respectively. Phenylpropanoids are induced by insect feeding and play a role in herbivore resistance^66,67^. Bd plants showed up-regulation of genes in all three pathways (Fig. 4). Transcripts for DEGs in phenylpropanoid biosynthesis, including chorismate mutase (CM), arogenate dehydratase (ADT), PAL, hydroxycinnamoyl-CoA shikimate/quinate hydroxycinnamoyl transferase (HCT), and cinnamate acid 4-hydroxylase (C4H) were strongly accumulated at all three time-points (Fig. 4, Supplementary Table S6). Similarly, DEGs encoding anthranilate synthase (AS), tryptophan synthase (TS), and indole-3-glycerol phosphate synthase (IGPS) in alkaloid biosynthesis via tryptophan were also up-regulated as early as 1 DAH. Key enzymes in terpenoid biosynthesis were also significantly expressed at all time-points. Thus, Hessian fly triggers the activation of key pathways leading to secondary metabolite biosynthesis in Bd. A similar increase in secondary metabolite-encoding gene expression is observed in resistant host wheat in response to Hessian fly larval attack^19^. These results implicate secondary metabolites as a key defensive measure in nonhost Bd against Hessian fly attack.

### Differential expression of transcription factors in Bd

Transcription factors (TFs) are important upstream regulators of stress-related genes resulting in activation or suppression of these genes, thereby imparting tolerance to a stress, including herbivory^68^. Stress-triggered TFs provide candidates for functional characterization to better understand how the perception of pests leads to resistance or susceptibility. Here, we identified 347 TF-encoding genes distributed over 26 families that responded to Hessian fly (Supplementary Fig. S7). Among these, highest number DEGs belonged to zinc finger proteins (136), followed by MYB-related genes (33), basic helix-loop-helix (bHLH, 31) Homeobox (HB, 27), WRKY (26), NAC (20), AP2-EREBP (19), basic leucine zipper domain (bZIP, 9), TEOSINTE BRANCED-1, CYCLOIDEA, PCF1 (TCP, 6), and 40 other TFs that were represented by less than 5 DEGs (Supplementary Fig. S7). While most genes encoding WRKY TFs were up-regulated, several of the DEGs encoding MYB and bHLH TFs were down-regulated (Supplementary Table S7). The WRKY TFs belong to a multigene family with several members playing a significant role in transcriptional reprogramming associated with plant immune response^69^. Several of these WRKYs may operate by either positively or negatively regulating Bd defense to Hessian fly, as documented in several other plant-pest interactions^70^. The highest up-regulated TF was WRKY55 (*Bradi2g22230*) and was up by 110-fold in Bd1, 40-fold in Bd3 and 26-fold in Bd5. In wheat lines susceptible to leaf rust pathogen, *Puccinia triticina*, TaWRKY55 was strongly up-regulated^71^ thus suggesting that WRKY55 may have a role as a negative regulator of resistance in Bd to Hessian fly. In addition, WRKY TFs are key regulators of secondary metabolites including phenylpropanoids, indole alkaloids and terpenoids^72^, which were up-regulated in Bd plants (Fig. 4). WRKY55 in *Hevea brasiliensis* regulates the production of polyterpene^73^. Up-regulated MYBs and bHLH TFs play a role in defense response against insects (aphids^74^, *Pieris rapae*^75^). However, in Bd, down-regulation of some of these TFs may be essential to modulate the plant immune signaling network, similar to whitefly-infested cotton plants^76^. Some DEGs encoding bZIP, NAC, AP2-EREBP and TRAF-like family proteins were highly up-regulated suggesting their role in regulating plant defense-response genes^77^. Several subclasses of the zinc finger TF family were over-represented in Bd though most of these DEGs were down-regulated. The zinc finger proteins are a super family of proteins known to regulate resistance mechanisms for various biotic stresses^78^. Zinc finger TFs were induced by *Spodoptera littoralis* feeding in *Arabidopsis* and played a JA-independent role in plant defense^79^. Similarly, transcripts for zinc finger TF, *StZFP2*, were induced in potato in response to infestation by tobacco hornworm (*Manduca sexta*) and Colorado potato beetle (*Leptinotarsa decemlineata*) implicating their role in plant defense against chewing insect pests^80^. However, in the case of Bd, this class of TFs may not be directly involved in plant defense.

### Hessian fly-associated signal transduction genes in Bd plants

**Figure 5:**
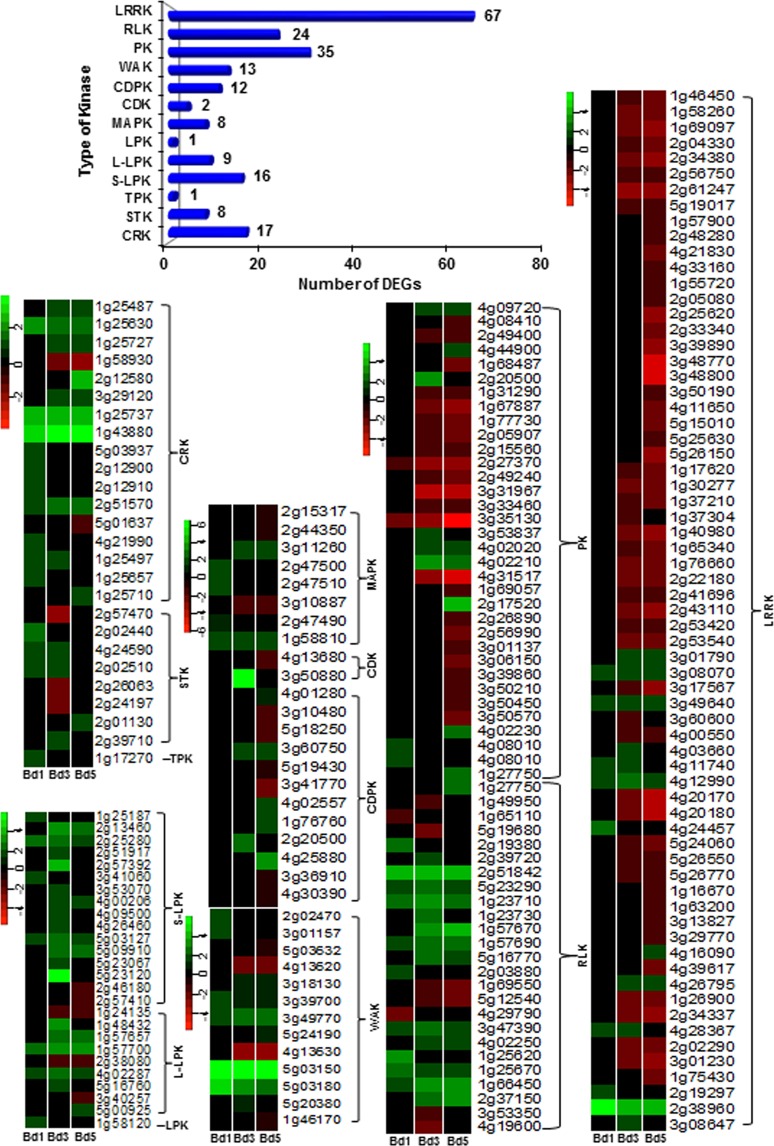
Differential expression of genes encoding protein kinases in *Brachypodium* infested with Hessian fly larvae. Number of DEGs encoding various types of protein kinases represented in the transcriptome of Hessian fly-infested Bd plants are indicated in the chart. Heatmaps depict expression profiles of DEGs encoding protein kinases over a time-course of 1 (Bd1), 3 (Bd3), and 5 (Bd5) DAH. All DEGs belonging to one type of protein kinase are clustered together within the heatmap. Since all genes represent the Bd Gene-IDs, the prefix “Bradi” has been removed and only the number associated with a particular Gene-ID is given for identification. Green represents up-regulated genes and red represents the down-regulated genes, while genes not differentially expressed at a particular time-point are indicated in black. CRK: cysteine-rich receptor-like kinase, STK: serine threonine protein kinase, TPK: tyrosine protein kinase, MAPK: mitogen-activated protein kinase, CDK: cyclin-dependent kinase, CDPK: calcium-dependent protein kinase, WAK: wall-associated kinase, S-LPK: s-locus lectin protein kinase, L-LPK: conA lectin-like kinase, LPK: lectin protein kinase, PK: protein kinase, RLK: receptor kinase/receptor-like kinase, LRRK: leucine rich repeat kinase.

Protein kinases play a central role in signal recognition and transduction leading to activation of plant defense mechanisms during biotic stress. In this study, multiple genes encoding protein kinases (213) were differentially expressed in Bd at all three time-points (Fig. 5). These were represented by cysteine-rich receptor-like kinases (CRK, 17), serine threonine protein kinases (STK, 8), tyrosine protein kinases (TPK, 1), mitogen-activated protein kinases (MAPK, 8), cyclin-dependent kinases (CDK, 2), calcium-dependent protein kinases (CDPK, 12), wall-associated kinases (WAK, 13), s-locus lectin protein kinases (S-LPK, 16), conA lectin-like kinases (L-LPK, 9), lectin protein kinase (LPK, 1), protein kinases (PK, 35), receptor-like kinases (RLK, 24), and leucine rich repeat kinases (LRRK, 67). Most genes encoding the lectin protein kinases (L-LPK, S-LPK, LPK) were up-regulated (Supplementary Table S8). Lectin protein kinases (LPK) are chimeric proteins with a lectin fused to extracellular kinase domains and are recognition systems at the cell surface, contributing to the detection of pathogens, and/or are involved in monitoring cell wall structure and cell growth^81^. Lectins are key defense proteins that play a significant role in defense against Hessian fly^17,18,30^. These lectins were also highly up-regulated in Bd in response to insect attack (Fig. 2). LPKs are vital for plant resistance to insects^82^. During herbivory by *M*. *sexta* larvae on tobacco (*Nicotiana attenuata*) plants, *lectin receptor kinase1* (*LecRK1*) suppressed insect-mediated inhibition of JA-induced defense responses, while decrease in *LecRK1* expression resulted in induced folivory as well as reduction of two key defense proteins, protease inhibitors and threonine deaminase^83^. Members from other kinase families including CRK, CDK, CDPK and RLK were also highly up-regulated in Bd. Interestingly, LRRK proteins that were represented with the largest number of DEGs were mostly down-regulated. Almost all of the genes encoding WAKs showed high transcript abundance with one gene (*Bradi5g03150*) encoding a WAK2 having transcripts as high as 39-fold (*p* < 0.0001) in Bd1 and increasing to almost 50-fold (*p* < 0.0001) in Bd3. In a highly resistant cultivar of cotton (*Gossypium hirsutum* cv HR) to whitefly (*Bemisia tabaci*), *WAK2* was down-regulated, while in the highly susceptible cultivar, ZS, the gene was up-regulated^76^. Thus, in Bd plants, *WAK2* may play a role in attempting to establish susceptibility.

### Photosynthetic pathway is suppressed in Bd plants

In response to galling insects, plants exhibit an inconsistent photosynthetic response, with some showing increased^84^ and others having decreased^85^ photosynthesis. Bd transcriptome revealed suppression of photosynthesis pathway genes (Supplementary Fig. S8). Most genes from both, the light-dependent and -independent reactions, were strongly down-regulated with the exception of four genes encoding chlorophyll a/b binding proteins, the photosystem II subunit R, and malate dehyrodgenase which showed a 2–6 fold increase in transcripts at Bd3 and Bd5 time-points, and one gene encoding NADP malic enzyme showing strong up-regulation (18-fold) in the Bd5 sample (Supplementary Table S9). Studies in resistant and susceptible host wheat following Hessian fly larval attack, showed increased photosynthesis only in the second leaf during resistance, and in the second and third leaf during susceptibility possibly due to altered source-sink relationships^86^. Down-regulation of photosynthesis-related genes is a common response to insect feeding in plants, representing a host defense mechanism^87^. Reduced photosynthesis in insect-attacked plants allows the redirection of resources like energy and carbon to produce defense-related metabolites^88^. It is possible that Bd plants channel carbon/nitrogen resources towards production of secondary metabolites to bolster a defense response.

### Cell wall integrity in Bd plants following larval attack

**Figure 6:**
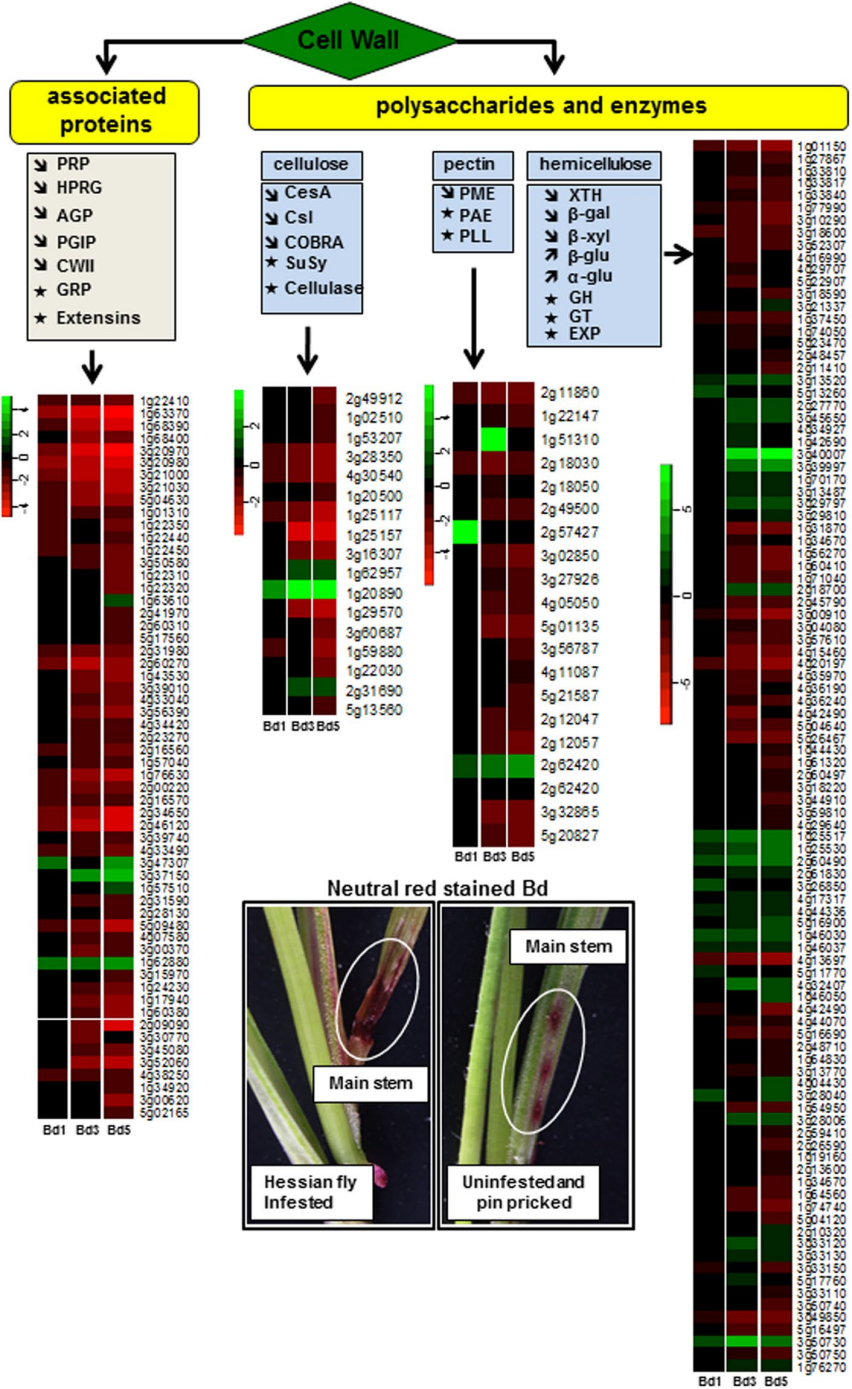
Cell wall metabolism in *Brachypodium* infested with Hessian fly larvae. Diagram showing list of genes involved in cell wall-related metabolism. Upward arrows indicate up-regulation and downward arrows indicate down-regulation of most members of a particular gene within the Hessian fly-infested Bd transcriptome. Asterisk represents a gene family with up- and down-regulated members. Heatmaps depict expression profiles of DEGs encoding cell wall-associated proteins and polysaccharides (cellulose, hemicellulose and pectins). The DEGs are grouped within a heatmap based on their associated function. Since all genes represent the Bd Gene-IDs, the prefix “Bradi” has been removed and only the number associated with a particular Gene-ID is given for identification. Green and red represent up-regulated and down-regulated genes, respectively, while genes not differentially expressed at a particular time-point are indicated in black. Neutral red stained Bd represents change in plant cell permeability in the main stem harboring the Hessian fly larvae at 4 DAH (left panel). Uninfested control Bd plants were pin pricked and stained with neutral red (right panel) to distinguish staining caused by larval feeding from that caused by physical damage. PRP: proline-rich protein, HPRG: hydroxyproline-rich glycoprotein, AGP: arabinogalactan protein, PGIP: polygalacturonase inhibiting protein, CWII: cell wall invertase inhibitor, GRP: glycine-rich protein, CesA: cellulose synthase, Csl: cellulose synthase-like, COBRA: glycosyl-phosphatidyl inositol-anchored protein, SuSy: sucrose synthase, PME: pectin methylesterase, PAE: pectin acetylesterase, PLL: pectin-lyase like, XTH: xyloglucan endotransglucosylase/hydrolase, β-gal: beta-galactosidase, β-xyl: beta-D-xylosidase, β-glu: beta-glucosidase, α-glu: alpha-glucosidase, GH: glycosylhydrolase, GT: glycosyltransferase, EXP: expansin.

The cell wall is a dynamic structure with the epidermal layer offering the first line of defense against biotic stress and often determining the outcome of interactions between plants and insects. Plants activate defense responses that lead to epidermal integrity and a dynamic cell wall remodeling to prevent a disease, while the insect employs strategies to break down this defense barrier^89^. In host wheat, Hessian fly larval salivary secretions induce epidermal cell permeability, as indicated by neutral red (NR) staining, initiating a two-way exchange of molecules modulating resistance and susceptibility^90^. This exchange leads to delivery of defense molecules during resistance and nutrients during susceptibility to the larvae^90^. To understand nonhost response, induced permeability was investigated by staining infested Bd plants with NR (Fig. 6). In the uninfested plants, the staining was localized to the manually punctured regions (Fig. 6), while in the Hessian fly-infested plants, NR staining was observed at the base of tiller 1, tiller 2 and main stem ranging in intensity scores from 0 to 7 as described previously^90^. The average intensity score on the main stem at the first time-point, 1 DAH, was 1.1 ± 0.3 (Supplementary Table S10); intensity increased to 2.6 ± 0.5 on 4 DAH (Fig. 6); and to 3.3 ± 0.4 by 8 DAH. In comparison to the previously documented NR staining in Hessian fly-infested host wheat^90^, Bd exhibited an intermediate level of staining between susceptible and resistant wheat. However, permeability in Bd did not continue to increase over time, as observed in susceptible wheat. To better understand the role of cell wall permeability in plant-insect interactions it is imperative to identify the function of wall components and how they interact in response to external stimuli. Typically, these components include cell wall polysaccharides (cellulose, hemicellulose, pectin) and other associated proteins^91^. Our study revealed a large repertoire of genes involved in cell wall metabolism in response to Hessian fly larval feeding (Fig. 6). Of the 58 cell wall-associated genes, 53 were strongly down-regulated while three encoding glycine-rich protein, one encoding extensin and one encoding hydroxyproline-rich glycoprotein showed a marginal increase in transcript abundance (Supplementary Table S11). Similarly, several enzymes involved in the synthesis of cell wall polymers (137) were significantly down-regulated (Fig. 6). Our results are comparable to expression profiles observed in susceptible host wheat where genes encoding enzymes involved in synthesis of cell wall components and loosening (wall extension and growth)^92^ were down-regulated^19^. Decreased expression of cell wall-related proteins and polymers is consistent with inhibition of fortification, thus creating a weaker cell wall that would facilitate increased insect feeding. Increased permeability by 4 DAH and lower expression of cell wall-associated genes in Bd plants suggests breakdown in the epidermal cell wall barriers allowing the larvae to effectively deliver salivary secretions to reprogram the plant into increasing production of nutrients^10,23^ and subsequent diffusion of nutrients to the leaf surface for larval consumption, as proposed during susceptible wheat-Hessian fly interactions^10^. However, persistent up-regulation of defense-related genes and secondary metabolites even as late as 5 DAH (Figs 2 and 4) may maintain a constant delivery of defense molecules to the epidermal layers, and repair or limit the cell wall damage, as evidenced by lower levels of permeability by 8 DAH compared to the susceptible wheat plants at the same stage^90^, thus preventing the larvae from getting substantial nutrients to complete their development. Hessian fly larval development seems to be arrested on Bd plants at varying stages, with 90% of the larvae dying and decomposing by 30 DAH, and a very few, severely under-developed larvae surviving till 46 DAH^5^.

## Conclusions

**Figure 7:**
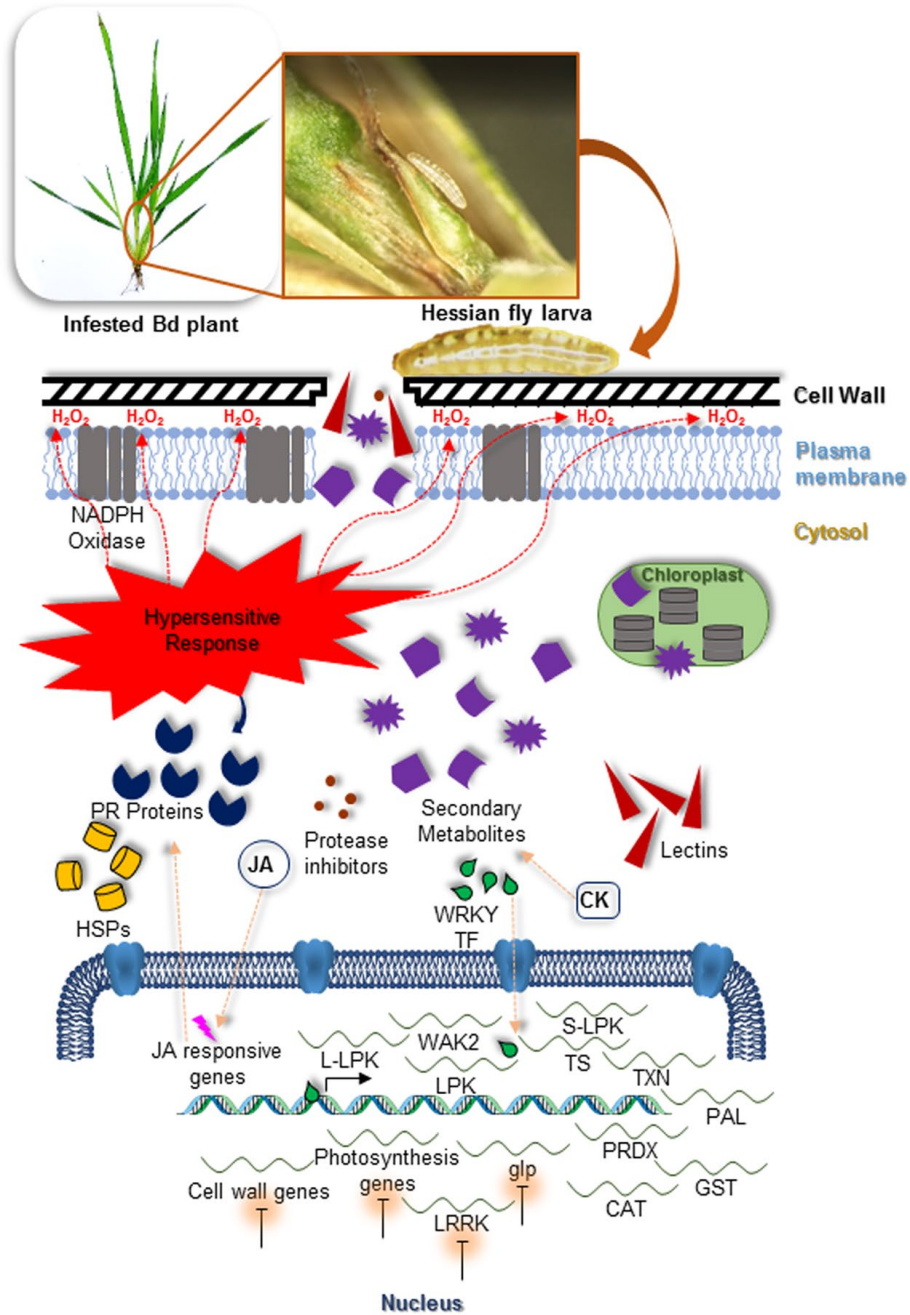
Model showing the major pathways involved during *Brachypodium*-Hessian fly interactions. Damage to cell wall and membrane by Hessian fly larval feeding triggers the production of a hypersensitive response resulting in the production of ROS such as H_2_O_2_ through the action of NADPH oxidase. Multiple defense strategies are mounted simultaneously. Jasmonic acid (JA)-responsive genes are induced leading to up-regulation of PR proteins. Various transcription factors (TF) such as WRKY trigger defense response genes such as different kinases, lectins, and protease inhibitors, while a number of photosynthesis and cell wall-associated genes are repressed resulting in delayed or suppressed cell wall fortification. Increased cytokinins (CK) induce secondary metabolite formation that may directly affect the survivability of the larvae. Some defense-responsive heat shock proteins (HSPs) play a role in resistance to the larvae, while some small HSPs induce susceptibility. WAK2: wall-associated kinase 2, S-LPK: s-locus protein kinase, L-LPK: conA lectin-like kinase, LPK: lectin protein kinase, LRRK: leucine rich repeat kinase, glp: germin-like protein, CAT: catalase, PRDX: peroxiredoxin, TXN: thioredoxin, GST: glutathione-S-transferase, TS: tryptophan synthase, PAL: phenylalanine ammonia lyase.

In conclusion, deciphering the global transcriptome expression profile of the Bd nonhost in response to Hessian fly larval feeding in the current study revealed the presence of competing defense strategies. Bd plants launched an early defense response on multiple fronts through the transcriptional activation of some classes of anti-pathogen TFs, initiation of ROS and a HR, production of insecticidal and antifeedant lectins, secondary metabolites, signaling molecules, and PIs potentially countering larval extra-oral salivary plant cell-degrading proteases (Fig. 7). These defense responses are similar to the ones seen in resistant host wheat against Hessian fly. At the same time, other molecular mechanisms comparable to those in susceptible host wheat are activated by the Hessian fly larval feeding. Among these responses were the early activation of certain TFs known to be associated with susceptibility and a suppression of other TFs that play a role in defense. In addition, at later time-points, sHSP and signal transduction genes associated with susceptibility were up-regulated along with up-regulation of CKs that potentially help establish nutrient centers for the larvae, and the down-regulation of genes involved in the fortification of cell walls as a barrier to the feeding larvae (Fig. 7). There was an extended expression of DR genes in Bd plants that temporally overlapped responses linked to susceptibility, and promoted intermediate physical and metabolic responses between resistant and susceptible phenotypes seen in host wheat. Our data reveal some of the molecular mechanisms that contribute to the ultimate battle for survival of a nonhost against an insect pest, and confirms the suitability of Bd as a model genome for future work involving functional studies of candidate Hessian fly-responsive genes that will aid in crop improvement strategies to increase resistance against this and other insect pests.

## Materials and Methods

### Insect and plant material

The Hessian fly (*Mayetiola destructor*) Biotype L stocks used in this study were maintained in diapause at 4 °C at the USDA-ARS Crop Production and Pest Control Research Unit in West Lafayette, IN, following the methods described by Sosa & Gallun^93^. *Brachypodium distachyon* seeds of line Bd21 were a gift from Roger Thilmony (Albany, USDA-ARS).

### Plant growth conditions and infestation

Ten Bd seeds were planted in 4-inch pots containing equal volume of vermiculite (Perlite Vermiculite Packaging Industries, North Bloomfield, OH) and Fafard professional potting mix (Conrad Fafard Inc., Agawam, MA) with a layer of Fertilome time-release fertilizer (19-6-12; Voluntary Purchasing Groups Inc., Bonham, TX). The pots were placed in a cold chamber for 1 week to ensure synchronized germination of all seeds. Pots were then transferred into a Conviron growth chamber (Controlled Environment Ltd., Winnipeg, Manitoba, Canada) set at 18 °C with 24 h photoperiod and 60% relative humidity. When plants reached the 3-leaf stage, they were infested with 10 female and 2 male Biotype L flies per pot as described previously^5^. Egg hatch and migration of Hessian fly larvae to the feeding site (crown of the plant) was monitored four days after infestation. A few pots containing Bd seedlings were left as uninfested controls.

### Tissue collection and RNA extraction

At the time of tissue harvest the plants were gently uprooted, the first leaf removed by peeling back, and 1.5 cm of the crown tissue above the root-shoot junction collected from infested (average 29 larvae per plant) and uninfested Bd plants. Tissue samples were collected 1, 3, and 5 days after egg-hatch (DAH) from the Hessian fly-infested Bd plants and are referred to as Bd1, Bd3 and Bd5. From the uninfested control Bd plants, tissue was harvested at 1 DAH time-point and is referred to as BdC. Tissues were collected from three biological replicates per treatment and time-point (20 plants per collection). Harvested tissues were frozen immediately in liquid nitrogen and stored at −80 °C. Total RNA was extracted from the frozen tissue using TRIzol reagent according to the manufacturer’s protocol (Life Technologies Corporation, Carlsbad, CA). RNA concentration was determined using a NanoDrop ND-1000 spectrophotometer (NanoDrop Technologies, Wilmington, DE).

### Illumina sequencing and data processing

RNA-sequencing (RNA-seq) library construction and sequencing was performed at the Purdue University Genomics Core Facility. Quality of extracted RNA was assessed using Agilent 2100 Bioanalyzer (Agilent Technologies, Santa Clara, CA) and RNA with integrity number (RIN) ≥ 8 was used for library preparation. The cDNA libraries were prepared using the TruSeq RNA Sample Prep Kit (Illumina Inc., San Diego, CA) as per manufacturer’s instructions. The libraries were then barcoded, pooled, and sequenced on HiSeq2500 instrument (Illumina Inc.).

Sequence quality for the filtered Illumina FASTq reads was assessed using FastQC (v 0.11.2) and quality trimming was done using FASTX toolkit (v 0.0.14) to retain high quality reads (Phred33 Score ≥30) and remove low quality reads. The quality trimmed reads were mapped against the bowtie2-indexed reference Bd genome^24^ using Tophat aligner (v 2.1.0). HTSeq (0.6.1) was used to generate raw read counts from each sample for each gene feature using Tophat output and the known Bd genome annotations. Counts from all three biological replicates were merged to generate a count matrix for all samples. Annotation for all the transcripts was done using BLAST homology search against the National Center for Biotechnology Information Non-Redundant (NCBI-NR) Protein database.

### Gene expression analyses

Merged counts matrix was used for downstream differential gene expression analyses. Pairwise comparisons were carried out between Hessian fly infested samples Bd1, Bd3, and Bd5 vs the uninfested control sample BdC. The analyses were done in ‘R’ (v 3.3.1) using three different methods: edgeR (v 3.14.0), voom (v 3.14.1), and DESeq2 (v 1.12.4). Differentially expressed genes (DEGs) detected in two or more methods were identified and paired with their official gene descriptions. Transcripts were called as differentially expressed when the False Discovery Rate (FDR) adjusted *p* values were <0.05 with log2 expression value >1 or <−1.

### Functional categorization and pathway analysis

The Protein Analysis Through Evolutionary Relationships (PANTHER) Classification System and analysis tools were used to categorize DEGs by Gene Ontology (GO) class of molecular function, biological process, and cellular component to determine if any of these classes or GO terms were overrepresented. To visualize the biotic stress biological pathways represented in the data, the DEGs were viewed with the MapMan biotic stress pathway analysis tool using Bd reference genome mapping file.

### Validation of RNA-seq gene expression

To validate the RNA-seq data, the expression profiles of 15 randomly selected up- and down-regulated genes were confirmed by quantitative reverse transcription-polymerase chain reaction (qRT-PCR) using LightCycler 480 Instrument II (Roche Diagnostics Corporation, Indianapolis, IN) as described previously^5^. Target-specific primers (Supplementary Table S12) were designed using Primer Express 3.0 (Applied Biosystems) software. The qRT-PCR results were obtained from three biological replicates with three technical replicates for each reaction. In addition, a Bd *Ubiquitin* gene was used as an endogenous control. Following amplification, primer specificity to a single target sequence was verified through melt-curve analysis. Relative expression levels of DEGs were determined using the Relative Standard Curve method (Applied Biosystems User Bulletin 2) as described previously^17^. Significant differences in the logarithm-transformed Relative Expression Values (REV) were determined by analysis of variance (ANOVA) using SAS as described previously^23^. The differences between infested and uninfested control samples were statistically significant if the *p* value associated with the contrast was <0.05.

### Assay for H_2_O_2_ accumulation

The DAB (3,3′-diaminobenzidine) staining method was used to detect H_2_O_2_ accumulation^94^ in Bd plants in response to Hessian fly larval attack. The Bd plants were grown and infested as described above. DAB staining solution was prepared by dissolving DAB powder (Sigma-Aldrich, St. Louis, MO) in deionized water (1 mg/mL) and the pH of the solution was adjusted to 3.8 with 0.2 N HCl. The DAB solution was kept in dark by covering with aluminum foil. At 1 DAH, six infested Bd plants were dissected under a microscope by cutting below the root/crown junction and the leaf sheaths were removed to expose the larval feeding sites on the main stem. Four uninfested control Bd plants were dissected in the same manner. Each plant was placed in a 15 mL falcon tube covered with aluminum foil. To each tube 10 mL of DAB solution was added, and vacuum infiltrated for 5 min. Following vacuum infiltration, the tubes were shaken at 100 rpm in a MaxQ 4000 Benchtop Orbital shaker (ThermoFisher Scientific, Waltham, MA) for 18 h at room temperature under dark. The plants were then photographed with a DP21 camera system on SZX2 stereomicroscope (Olympus America Inc., Center Valley, PA).

### Staining to assess cell wall permeability

To determine whether the Hessian fly larvae disrupt the integrity of the epidermal cell layer, Bd plants were stained with NR to assess permeability as described in Williams *et al*.^90^. Bd plants were grown and infested as described above. At 1, 4, and 8 DAH, 10 infested plants were dissected by cutting below the root/crown junction and peeling back the leaves exposing the main stem and tiller leaves. Care was taken to avoid wounding the plants during the dissection process. Plants were placed in 0.1% NR (Sigma-Aldrich) stain for 10 min, then rinsed thoroughly with water. Uninfested plants were also dissected and stained as negative controls or were poked with a 0.2 mm minuten pin prior to staining as positive controls. Intensity of red staining was scored according to the scale used in Williams *et al*.^90^ with a score of 0 indicating no stain and a score of 7 indicating a completely red crown. Following staining, photomicrographs were taken with a DP21 camera system on SZX2 stereomicroscope (Olympus).

## Supplementary information

Supplementary_Figures

Supplementary Table S1

Supplementary Table S2

Supplementary Table S3

Supplementary Table S4

Supplementary Table S5

Supplementary Table S6

Supplementary Table S7

Supplementary Table S8

Supplementary Table S9

Supplementary Table S10

Supplementary Table S11

Supplementary Table S12
