# Supplementary material for: Multiple molecular defense strategies in Brachypodium distachyon surmount Hessian fly (Mayetiola destructor) larvae-induced susceptibility for plant survival
Source: Sci Rep. 2019 Feb 22;9:2596. doi: 10.1038/s41598-019-39615-2 (PMC6385206; doi:10.1038/s41598-019-39615-2)
Supplement: Supplementary file 1 — Supplementary_Figures [file 41598_2019_39615_MOESM1_ESM.pdf]

Multiple molecular defense strategies in *Brachypodium distachyon* surmount Hessian fly (*Mayetiola destructor*) larvae-induced susceptibility for plant survival

Subhashree Subramanyam[1,*], Jill A. Nemacheck[2], Andrea M. Hargarten[2], Nagesh Sardesai[3], Brandon J. Schemerhorn[2,4], Christie E. Williams[1,2]

[1]Department of Agronomy, Purdue University, West Lafayette, IN 47907

[2]USDA-ARS Crop Production and Pest Control Research Unit, West Lafayette, IN 47907 [3]Corteva Agriscience™, Agriculture Division of DowDuPont, Johnston IA 50131 [4]Department of Entomology, Purdue University, West Lafayette, IN 47907

[*]Correspondence and requests for materials should be addressed to S.S. (email: shubha@purdue.edu)

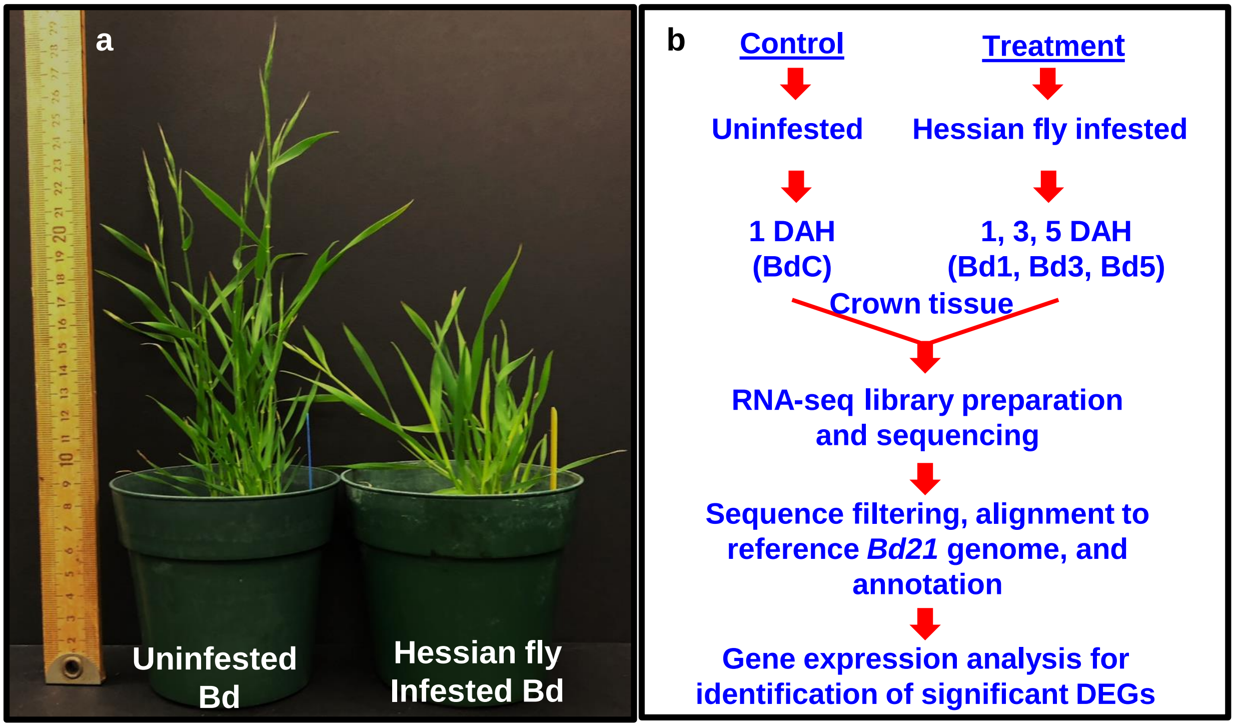

Supplementary Figure S1. Experimental material and design. (**a**) Hessian fly-infested and uninfested *Brachypodium distachyon* (Bd) crown tissue was used as the experimental material for RNA-seq transcriptome analyses. (**b**) Schematic representation of experimental design for RNA-seq study. DEGs (differentially expressed genes) were identified by comparing the mRNA abundance of transcripts in Hessian fly-infested Bd plants at 1 (Bd1), 3 (Bd3) and 5 (Bd5) Days After Egg-Hatch (DAH) to the uninfested control plants at 1 DAH (BdC) using RNA-seq and a 2-fold change cut-off $p < 0.05$).

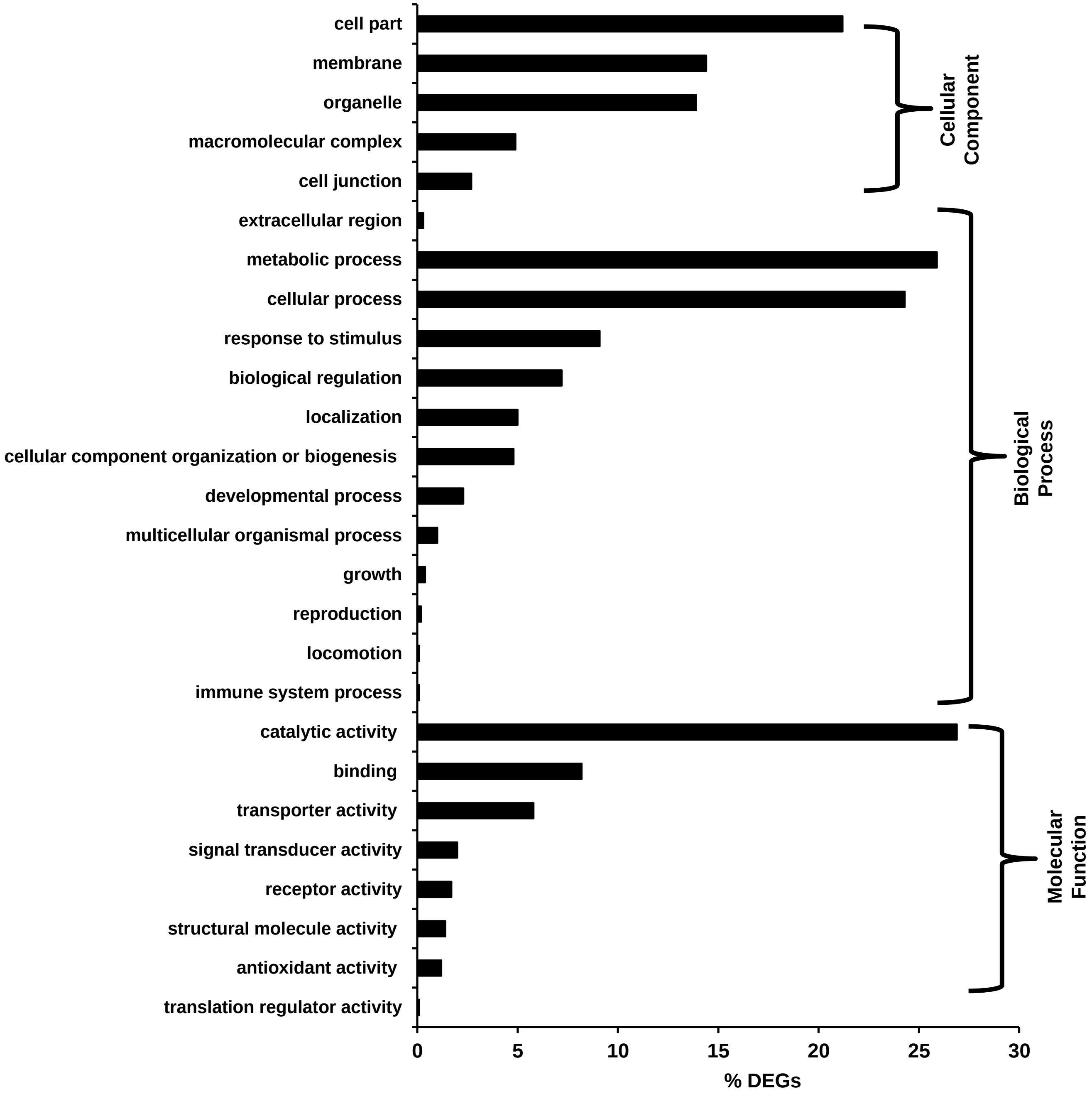

Supplementary Figure S2. Gene Ontology (GO) enrichment analysis of DEGs. Functional categorization of up- and down-regulated DEGs identified by RNA-seq in Bd1, Bd3 and Bd5 samples based on GO annotations. The X-axis shows the percent DEGs and Y-axis represents the GO categories classified as cellular component, biological process and molecular functions.

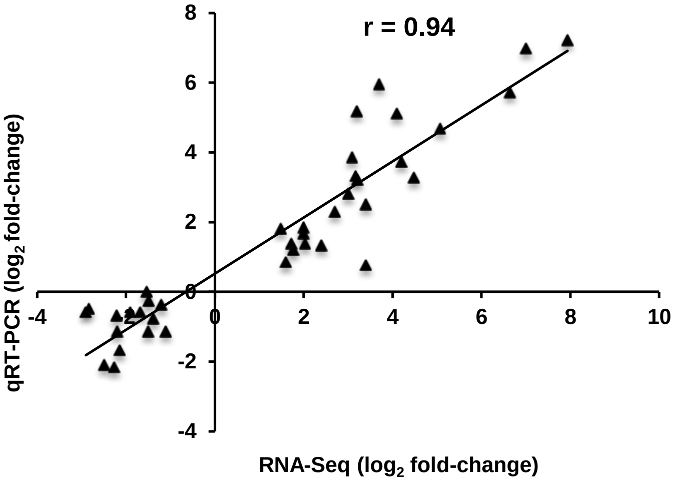

Supplementary Figure S3. Correlation between RNA-seq and qRT-PCR expression data. Pearson correlation coefficient (r=0.94) was used to determine the similarity in gene expression pattern between RNA-seq and qRT-PCR for 15 (9 up-regulated and 6 down-regulated) select DEGs. All expression data were normalized in log2 scale.

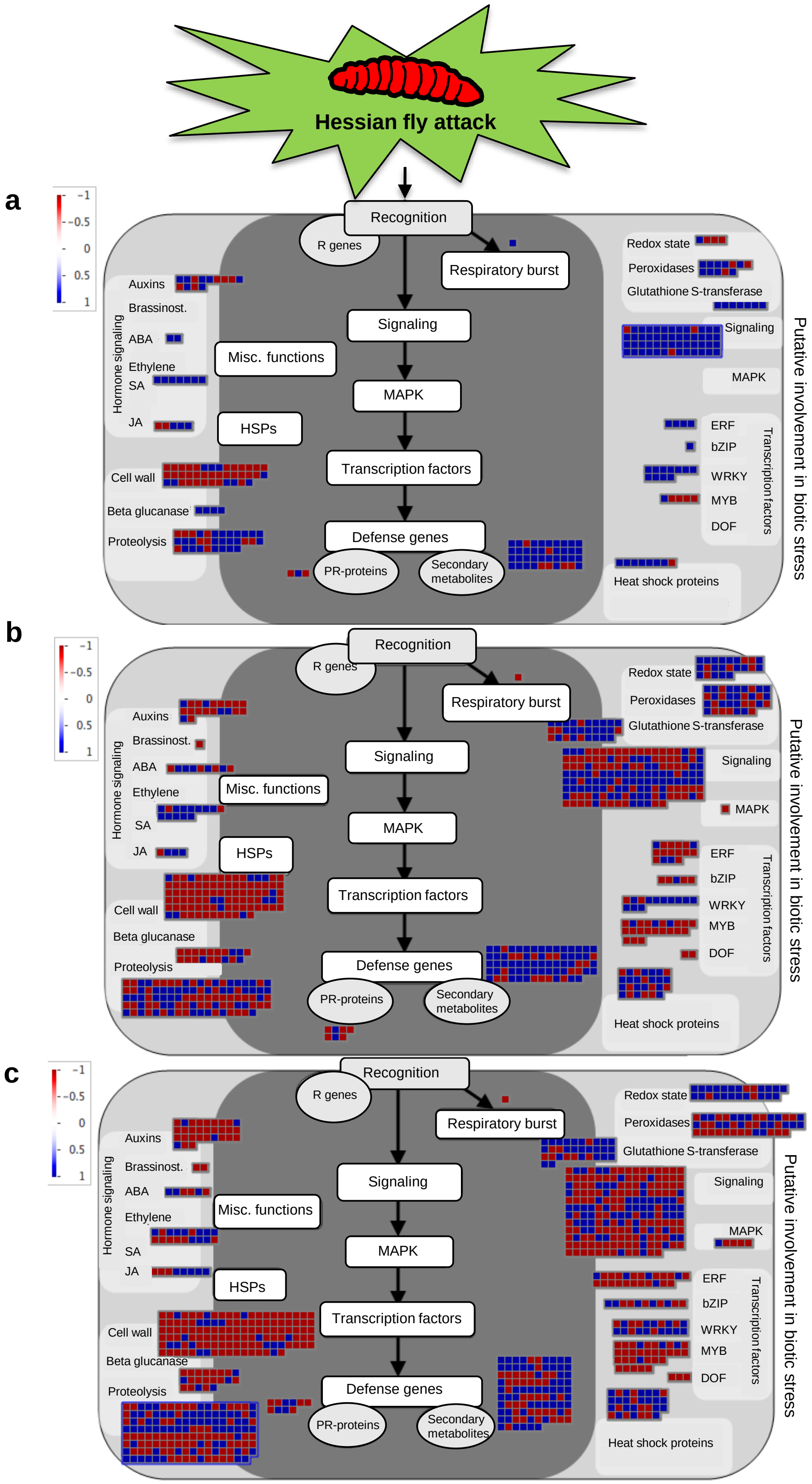

Supplementary Figure S4. DEGs involved in biotic stress pathways in *Brachypodium*. MapMan pathway analysis revealed biotic stress pathway overviews of statistically significant (log2 fold change, $p < 0.05$) DEGs in Hessian fly-infested Bd plants over a time-course (**a**) Bd1 (1DAH), (**b**) Bd3 (3DAH), and (**c**) Bd5 (5 DAH). Blue and red represent up- and down-regulated genes, respectively.

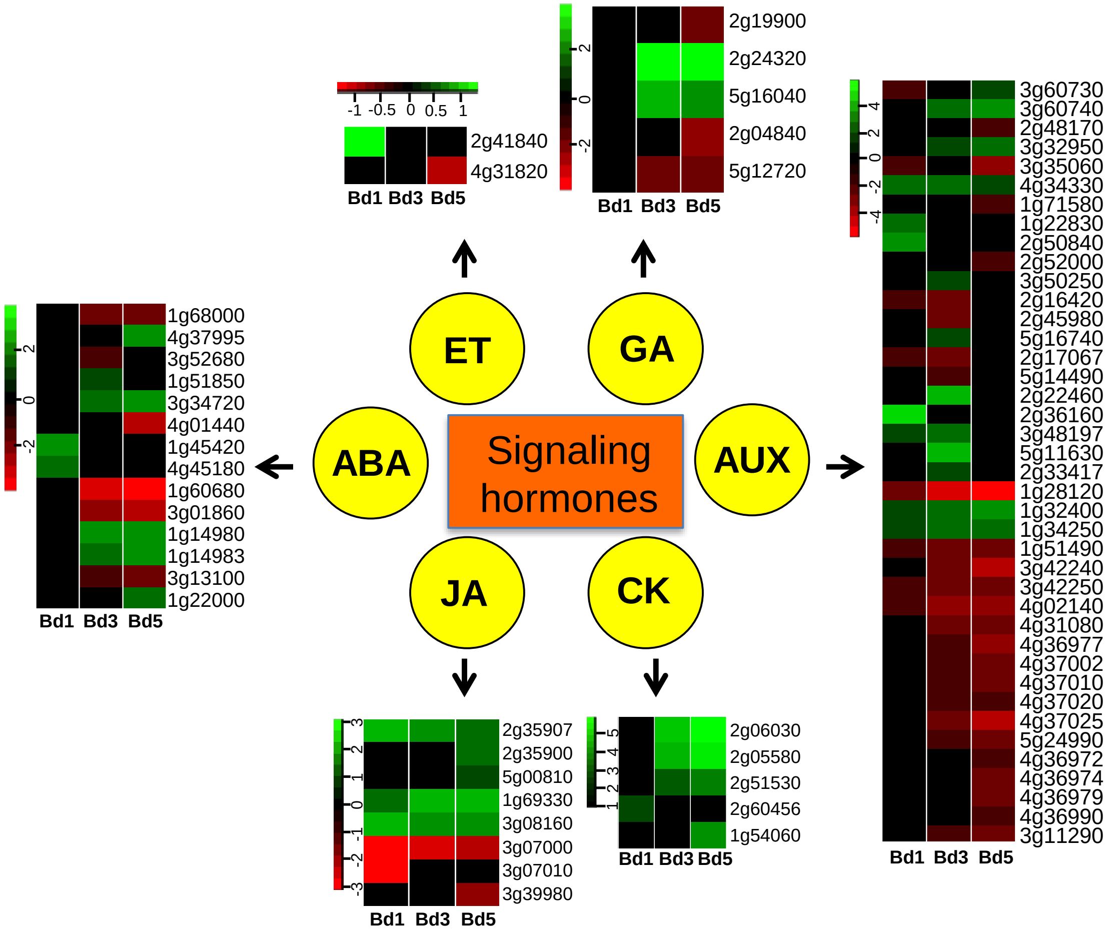

Supplementary Figure S5. Differential regulation of *Brachypodium* genes encoding signaling hormones in response to Hessian fly larval feeding. Heatmaps depict expression profiles of DEGs involved in signaling hormone pathways over a time course of 1 (Bd1), 3 (Bd3), and 5 (Bd5) DAH. All DEGs encoding a particular signaling hormone are clustered together within a heatmap. Since all genes represent the Bd Gene-IDs, the prefix "Bradi" has been removed and only the number associated with a particular Gene-ID is given for identification. Green represents up-regulated genes and red represents down-regulated genes, while genes not differentially expressed at a particular time-point are indicated in black. ABA: abscisic acid, ET: ethylene, GA: gibberellic acid, JA: jasmonic acid, AUX: auxins, CK: cytokinins.

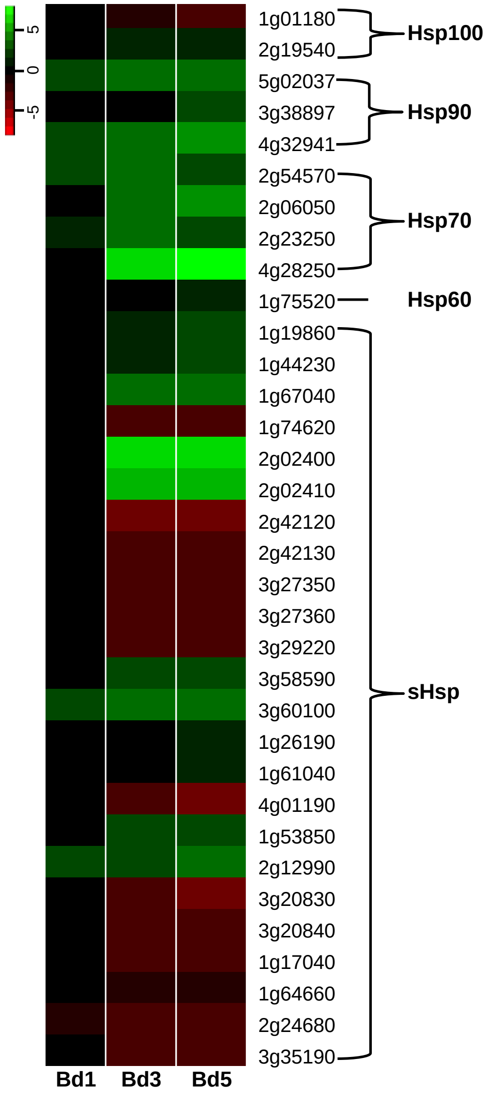

Supplementary Figure S6. Differential expression of *Brachypodium* genes encoding heat shock proteins in response to Hessian fly larval feeding. Heatmap depicts genes encoding differentially expressing HSPs over a time course 1 (Bd1), 3 (Bd3), and 5 (Bd5) DAH, and grouped based on the five HSP families (HSP100, HSP90, HSP70, HSP60 and small HSP) they represent. Since all genes represent the Bd Gene-IDs, the prefix "Bradi" has been removed and only the number associated with a particular Gene-ID is given for identification. Green represents up-regulated genes and red represents the down-regulated genes, while genes not differentially expressed at a particular time-point are indicated in black.

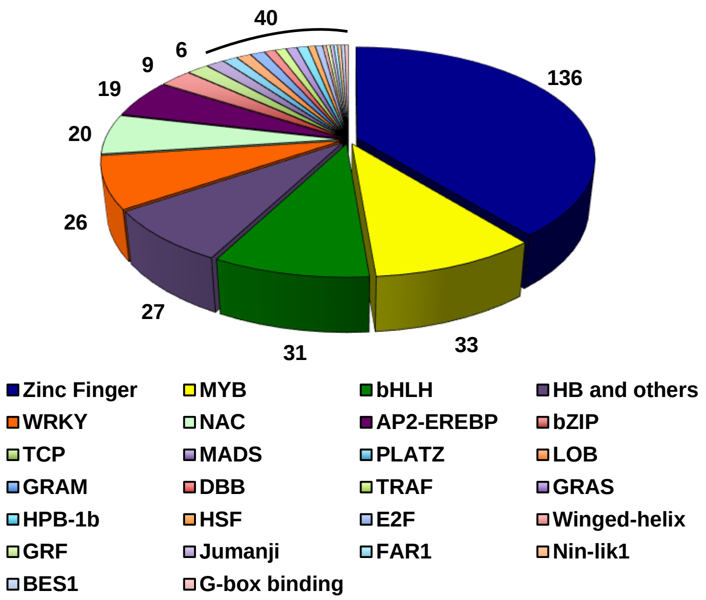

Supplementary Figure S7. Differential expression of transcription factors in *Brachypodium* infested with Hessain fly larvae. Pie chart showing the different classes and number of DEGs encoding transcription factors belonging to different families represented in the Hessian fly-infested Bd transcriptome.

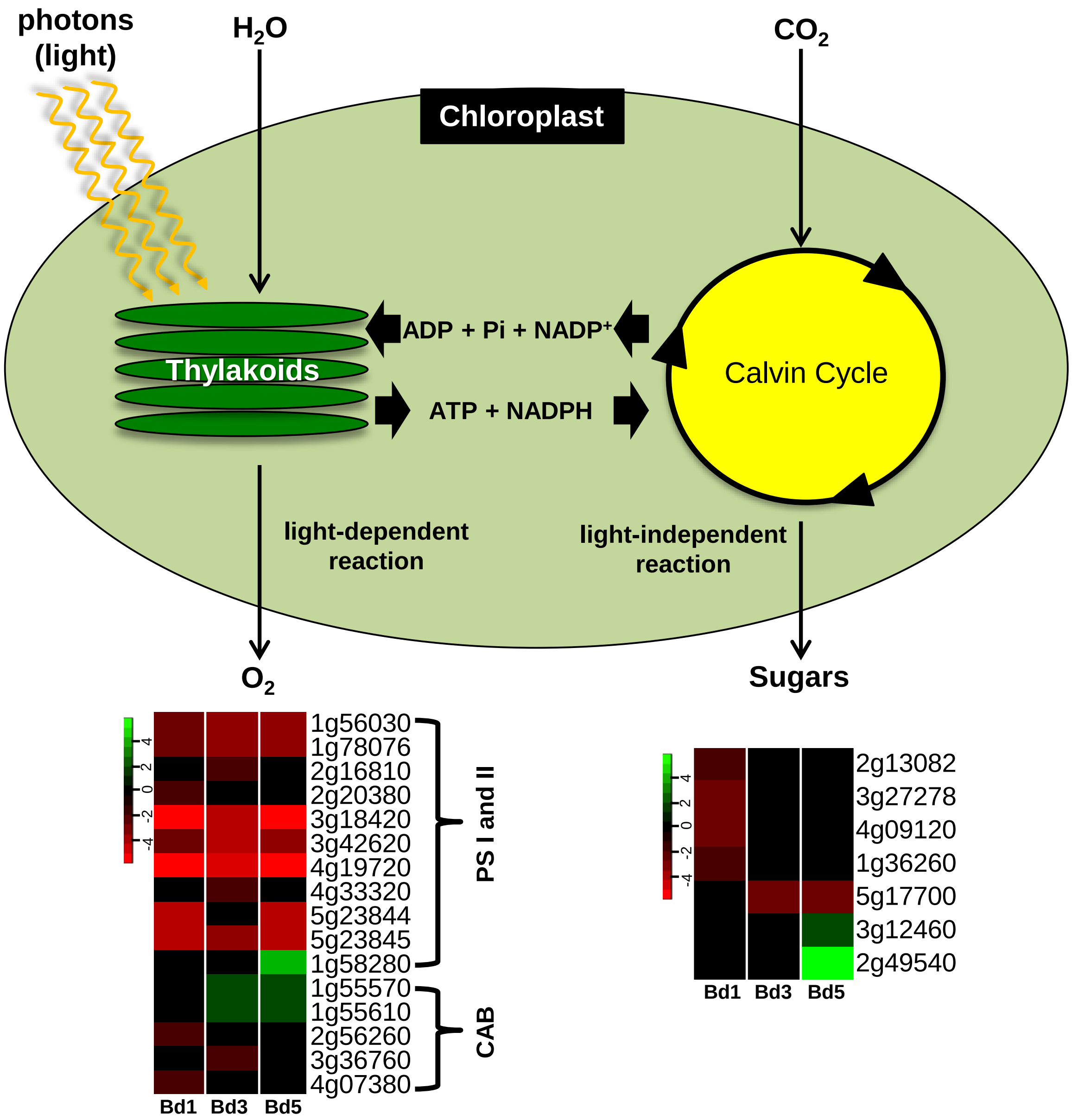

Supplementary Figure S8. Differential expression of *Brachypodium* genes encoding enzymes involved in photosynthesis during Hessian fly infestation. Schematic representation of the chloroplast-localized photosynthetic pathway in plants, converting water (H_2O) and carbon dioxide (CO_2) in the presence of photons (light) to oxygen (O_2) and sugars. Heatmaps depict DEGs encoding enzymes involved in light-dependent and Calvin cycle (light-independent) photosynthetic pathways in Hessian fly-infested Bd plants over a time course of 1 (Bd1), 3 (Bd3), and 5 (Bd5) DAH. Since all genes represent the Bd Gene-IDs, the prefix "Bradi" has been removed and only the number associated with a particular Gene-ID is given for identification. Green represents up-regulated and red represents down-regulated genes, while genes that are not differentially expressed at a particular time-point are indicated in black. PS I and II: Photosystem I and II proteins, CAB: Chlorophyll a/b binding proteins.
