# Supplementary material for: Multiple molecular defense strategies in Brachypodium distachyon surmount Hessian fly (Mayetiola destructor) larvae-induced susceptibility for plant survival
Source: Sci Rep. 2019 Feb 22;9:2596. doi: 10.1038/s41598-019-39615-2 (PMC6385206; doi:10.1038/s41598-019-39615-2)
Supplement: Supplementary file 2 — Supplementary Table S1 [file 41598_2019_39615_MOESM2_ESM.docx]

**Supplementary Table S1.** qRT-PCR validation of RNA-seq expression results

|  |  | **RNA-Seq**** | | | **qRT-PCR**** | | |
| --- | --- | --- | --- | --- | --- | --- | --- |
| **Gene-ID*** | **Function** | **Bd1** | **Bd3** | **Bd5** | **Bd1** | **Bd3** | **Bd5** |
| 3g28350 | cellulose synthase | -2.1 | -2.8 | -4.6 | -2.2 | -2.2 | -2.2 |
| 1g26920 | MYB domain | -2.3 | -3.7 | -7.5 | -1.3 | -1.5 | -1.5 |
| 2g24680 | heat shock protein DnaJ | -2.6 | -4.4 | -5.6 | -1.7 | -3.2 | -4.3 |
| 3g42620 | photosystem II | -2.9 | -7.1 | -4.6 | - | -1.4 | -1.6 |
| 3g59320 | TCP transcription factor | - | -2.8 | -3.2 | - | -1.2 | -1.5 |
| 2g57317 | mildew resistance locus O 1 | - | - | -4.8 | - | - | -4.5 |
| 3g43160 | cinnamate-4-hydroxylase | 10.6 | 3.0 | 4.0 | 1.7 | 1.8 | 3.2 |
| 2g54570 | heat shock protein 70 | 4.0 | 9.0 | 6.5 | 3.6 | 10.0 | 4.9 |
| 2g22230 | WRKY41 | 100.3 | 33.7 | 22.4 | 52.8 | 25.6 | 9.7 |
| 2g24820 | BBTI11 | 5.3 | 10.6 | 18.4 | 2.5 | 5.7 | 13.2 |
| 3g15956 | terpene synthase | 9.2 | 17.1 | 13.0 | 36.2 | 34.5 | 62.0 |
| 5g27130 | peroxidase precursor | 8.0 | 9.3 | 8.5 | 7.0 | 9.2 | 14.4 |
| 2g25280 | S-locus protein kinase | 4.1 | 3.3 | 2.8 | 2.6 | 2.6 | 3.5 |
| 1g20040 | 1-cysteine peroxiredoxin 1 | - | 128.6 | 244.9 | - | 126.2 | 148.7 |
| 1g64970 | senescence associated gene 20 | 3.4 | - | - | 2.3 | - | - |

* Since all genes represent the Bd Gene-IDs, the prefix “Bradi” has been removed and only the number associated with a particular Gene-ID is given for identification

** Data shown as fold-change of mRNA abundance in Hessian fly-infested crown tissue samples at 1 (Bd1), 3 (Bd3), and Bd5 (5) days after egg-hatch compared to the uninfested Bd control (BdC) tissue (*p*<0.05)
