# Supplementary material for: Multiple molecular defense strategies in Brachypodium distachyon surmount Hessian fly (Mayetiola destructor) larvae-induced susceptibility for plant survival
Source: Sci Rep. 2019 Feb 22;9:2596. doi: 10.1038/s41598-019-39615-2 (PMC6385206; doi:10.1038/s41598-019-39615-2)
Supplement: Supplementary file 11 — Supplementary Table S10 [file 41598_2019_39615_MOESM11_ESM.docx]

**Supplementary Table S10.** Neutral Red scoring* of Hessian fly-infested Bd plants.

| **Plant#** | **1 DAH** | **4 DAH** | **8 DAH** |
| --- | --- | --- | --- |
| **1** | 0 | 3 | 5 |
| **2** | 1 | 3 | 0 |
| **3** | 3 | 4 | 4 |
| **4** | 3 | 4 | 3 |
| **5** | 0 | 4 | 1 |
| **6** | 0 | 3 | 4 |
| **7** | 1 | 4 | 4 |
| **8** | 1 | 0 | 4 |
| **9** | 0 | 0 | 4 |
| **10** | 2 | 1 | 4 |
| **Average** | **1.1±0.3** | **2.6±0.5** | **3.3±0.4** |
|  |  |  |  |

*At each time-point, ten plants were dissected to expose the feeding sites, stained with Neutral Red, and the intensity of red stain was scored on a scale of 0-7, according to Williams et al., 2011. Each individual plant score is shown along with the average score and standard error. Intensity increased between 1 DAH and 4 DAH, but did not change significantly between 4 DAH and 8 DAH.
