# Supplementary material for: Multiple molecular defense strategies in Brachypodium distachyon surmount Hessian fly (Mayetiola destructor) larvae-induced susceptibility for plant survival
Source: Sci Rep. 2019 Feb 22;9:2596. doi: 10.1038/s41598-019-39615-2 (PMC6385206; doi:10.1038/s41598-019-39615-2)
Supplement: Supplementary file 13 — Supplementary Table S12 [file 41598_2019_39615_MOESM13_ESM.docx]

**Supplementary Table S12**: Gene-specific primer sequences for validation of RNA-seq expression data by qRT-PCR

**________________________________________________________________________**

**Gene-ID* Description Forward Primer Reverse Primer**

3g28350 CESA CACACATCGCACGTTTTGG CCTCGCTCGCAAAACTTGTAC

1g26920 MYB domain TTCCTGTCCTTCTTCATTCAGAATT TCACTCAGCTATGCATTTCTTGCT

2g24680 Hsp DnaJ GACGGGCCTCGCTGAGAT CATGGCACCGACAAAATTTACTC

3g42620 photosystem II GCTGAGCTCAAGGTGAAAGAGATC AACCCGAACATGGAGAACATG

3g59320 TCP transcription factor GCGCGTGCATTGGTCTCT CCAACAAATTAAGAACAGATCAAAGC

2g57317 MLO1 GGATCTCCTTCCTTCCCCTTCT TGACGTGCTCCAGCTTGGT

3g43160 C-4-H AGAGAAAAGAAGGAAGGTGATGGA TCGCGCATCGGAGCTT

2g54570 heat shock protein 70 GCGGTGTTTGATTGTTTTGGT TTCAACGACACGATACTGCATAGTT

2g22230 WRKY41 transcription factor TCTTCGTCCTCGCTACCATTG TGACTTCATCTCCATTTCCATCTC

2g24820 Bowman Birk Inhibitor 11 TCGTTGTATTGTCACCGTGTTTG CGATCGGCACTGATTTTTATTTT

3g15956 terpene synthase GACCACGGGACGACAAAGG TCCATGAGTACTCGGCAAGTTCT

5g27130 peroxidase precursor CCTGGGTGCCCGTAAGC TTGCGGCGTCCATGGT

2g25280 S-locus protein kinase CAAATGGCGGAACATCAAGAT AGCGATCACGAAGCAGAAGAC

1g20040 1-cysteine peroxiredoxin 1 GGTGGGAGAAGAGGATGACGTA TGCGGCAGGATCTGCAT

1g64970 senescence-associated gene 20 GCTGCGATCGATCCTGTGTA GTCGGGAGCTCATCTCATCAG

4g00660** ubiquitin-conjugating enzyme18 CGACTTCCCCGAGCATTATC GCCGGGTTGAGGAAAATGA

* Since all genes represents Bd Gene-IDs, the prefix “Bradi’ has been removed and only the number associated with a particular Gene-ID is given for identification.

** Gene used as endogenous control
